# Impact of staging on survival outcomes: a nationwide real-world cohort study of metastatic uveal melanoma

**DOI:** 10.1097/CMR.0000000000000728

**Published:** 2021-03-05

**Authors:** Elina S. Rantala, Tero T. Kivelä, Micaela M. Hernberg

**Affiliations:** a Ocular Oncology Service, Department of Ophthalmology, University of Helsinki and Helsinki University Hospital; b Comprehensive Cancer Center, Department of Oncology, Helsinki University Hospital and University of Helsinki, Helsinki, Finland

**Keywords:** cohort studies, Kaplan–Meier estimate, melanoma, metastasis, staging, survival, treatment, uveal melanoma, uveal neoplasms

## Abstract

No data exist regarding whether any first-line treatment for metastatic uveal melanoma provides overall survival (OS) benefit, if staged and compared to best supportive care (BSC). We analyzed OS in a nationwide, consecutive cohort diagnosed with metastatic uveal melanoma between January 1999 and December 2016. The Helsinki University Hospital Working Formulation was used to assign patients to stage IVa, IVb and IVc, corresponding to predicted median OS ≥12, <12–6 and <6 months, respectively. OS of 216 actively treated patients was compared by treatment and working formulation stage against 108 similarly staged, concurrent patients managed with BSC using Kaplan–Meier analysis and Cox regression. The median OS with active treatment was 18 (range, 0.7–162), 6.9 (range, 1.3–30) and 1.9 (range, 0.2–18) months in working formulation stage IVa, IVb and IVc, respectively. Patients who received chemoimmunotherapy, selective internal radiation therapy, or underwent surgical resection survived longer – median OS 13, 16 and 24 months, respectively – than those receiving conventional chemotherapy – median OS 5.1 months – but only with surgical resection their OS exceeded that with BSC, both overall and in stage IVa (*P* < 0.001, *P* = 0.010). In stage IVb and IVc, no difference in OS was observed in any comparison. Staging of patients is crucial when comparing survival after metastatic uveal melanoma. Only surgical resection for stage IVa disease provided longer OS in our national cohort. We additionally recommend stage-specific comparison of novel treatments against available BSC data.

## Abstract 2

Supplemental Digital Content is available in the text.

## Introduction

More than half of patients with primary uveal melanoma develop metastases [1,2]. The liver is the most common first site, and local treatments have been suggested to prolong [3–6] the otherwise limited median overall survival (OS) of approximately 13 months [1,7]. Most studies have been small, noncomparative and often retrospective [1,7]. Randomized trials have been rare [8–13]. The largest one found among 171 patients no difference in OS between intravenous and intra-arterial fotemustine, a chemotherapeutic agent that concentrates in the liver [8].

Five recent surveys report broader real-life outcomes. Only one registry study of 175 patients of whom 106 received active treatment was nationwide [14]. The other four were cohorts from single tertiary referral centers with 62–539 actively treated patients [4,6,15,16]. The key limitation common to them was that patients were not staged and often lacked a proper control group. For these reasons, the influence on survival of prognostic factors cannot be judged, especially not by treatment modality. To take a step forward, we report OS by validated stages [17] in a nationwide cohort of consecutive patients with newly diagnosed metastatic uveal melanoma and compare stage-specific OS by treatment and against best supportive care (BSC).

## Methods

### Aims of the study

Our primary aim is to report population-based OS of actively treated patients with metastatic uveal melanoma stratified by validated prognostic stages [17] by treatment type. Our secondary aim is to compare the stage-specific OS to our similarly staged BSC cohort [18].

### Study design

Eligible to our retrospective observational cohort study were patients treated for primary uveal melanoma in the Ocular Oncology Service, Department of Ophthalmology, Helsinki University Hospital, Finland, a national referral center managing over 95% of uveal melanoma in Finland, who were diagnosed with metastases between 1 January 1999 and 31 December 2016. The institutional review board and the National Institute for Health and Welfare approved the study. Informed consent was not required by Finnish law because the study was based on past patient records. Exclusion criteria were adjuvant therapy after treatment of the primary, concurrent active second cancer and absence of treatment details.

### Data collection

We obtained patient charts from all hospitals that participated in management and grouped patients according to first-line therapy: active treatment or BSC. Of 338 patients, 113 were not actively treated (see Supplementary Figure S1, Supplemental digital content 1, http://links.lww.com/MR/A258 which contains the flow chart). Data on 108 patients who received BSC, available at https://doi.org/10.5281/zenodo.3369090 [18], were analyzed identically to those receiving active treatment. Nine of 225 actively treated patients were excluded: one received adjuvant therapy, three had an active second cancer [18] and records had been destroyed for five patients. Data of 11 of the remaining 216 patients were partial because the law permits discarding most patient records 12 years after death.

We recorded gender, age, date of diagnosis of primary uveal melanoma and its metastases, American Joint Committee on Cancer 8th edition tumor, node, metastasis (TNM) staging [19,20] and participation in annual follow-up [21] with liver function tests (LFTs) and upper abdominal ultrasonography to detect metastases (semiannually from 2014 onward for TNM stage III), followed by staging computed tomography, magnetic resonance imaging, or both, when metastases were suspected. Furthermore, we recorded serum or plasma levels of LFTs, sites of metastases, the largest diameter of the largest metastasis (LDLM), symptoms from metastases, Eastern Cooperative Oncology Group performance status [22] at the time of treatment decision, treatment modality and the date and cause of death. Follow-up ended on 31 December 2018. Median follow-up time was 3.8 years (range, 0.1–24).

### Verification of metastases

We adapted definitions of the Collaborative Ocular Melanoma Study [1,23] to assess the level of evidence for metastatic uveal melanoma, and reviewed histopathological specimens as required (see Supplementary Text S1, Supplemental digital content 2, http://links.lww.com/MR/A259 that describes the verification of metastases). Seventy-one percent of metastases were coded as confirmed, 7% as suspected and 2% as possible, whereas 20% had not been biopsied and were diagnosed with imaging.

### Staging of metastases

TNM staging divides metastatic uveal melanoma in three categories (M1a to M1c) by LDLM [19]. In addition to LDLM, performance status and serum or plasma alkaline phosphatase level are independent predictors of survival and, consequently, we used the Helsinki University Hospital Working Formulation staging that includes all three variables (see Supplementary Table S1, Supplemental digital content 2 http://links.lww.com/MR/A259 that illustrates the categorization of stage IV uveal melanomas according to working formulation) [24]. It has been validated by the European Ophthalmic Oncology Group [17] and enables calculation of predicted median OS (online calculator available at http://www.prognomics.org/huhwf.aspx). We used data at the time of treatment decision to assign patients to working formulation stage IVa, IVb and IVc, corresponding to median predicted OS of ≥12, <12–6 and <6 months, respectively. Performance status, LDLM, or alkaline phosphatase level was missing from 12 patients, but we could assign stage for nine of them by using the published prognostic table [24].

### Treatment categories

Based on our previous meta-analysis [7], we prospectively identified the following systemic treatment modalities: conventional chemotherapy, chemoimmunotherapy, checkpoint inhibitors (CPI), protein kinase inhibitors (PKI) and vaccine therapies. Additionally, we had data on interferon/interleukin (IFN/IL) monotherapy. Prospectively identified local treatments were surgery, selective internal radiation therapy (SIRT), trans-arterial chemoembolization (TACE) and other liver-directed therapies (LDT; stereotactic radiofrequency ablation and brachytherapy), all first-line [7].

### Data on best supportive care

Briefly, the median OS of the 108 patients was 1.6 (range, 0–83) months, and 24, 19, and 55% of them were assigned to working formulation stage IVa, IVb and IVc, respectively [18]. The corresponding median stage-specific OS was 12 (range, 1.6–83), 5.7 (range, 0.5–40) and 0.6 (range, 0–8.0) months from treatment that is BSC decision [17,18].

### Statistical analysis

Analysis was performed with Stata (version 16, Stata, College Station, Texas, USA). Significance was set at <0.05. All *P* values were two-tailed. We report median with range and interquartile range (IQR) for continuous variables. Primary endpoint was OS from treatment decision to death, as is most common in clinical trials and required of trials by the European Medicines Agency and the US Food and Drug Administration [7,25,26]. We compared first-line treatment modalities received by at least 10 patients [27], systemic vs. local treatment if only hepatic metastases were detected, and used stage-specific OS with BSC as reference. The number of treatment lines and modalities were recorded.

We estimated OS using the Kaplan–Meier product-limit method, report median OS with 95% confidence interval (CI), and compared unordered and ordered categories with the log-rank test and test for trend, respectively. We adjusted with Bonferroni correction for family-wise multiple comparisons. We used Cox proportional hazards regression to explore whether additional variables might independently contribute to predicting OS, given the working formulation stage. We allowed independent variables in models if *P *<* *0.10, tested the assumption of proportional hazards using scaled adjustment of Schoenfeld residuals [28], and compared nested models using the deviance test.

## Results

Of the 216 actively treated patients with metastatic uveal melanoma, 49% were female, 99% attended regular follow-up with upper abdominal ultrasonography and LFTs and 76% were asymptomatic (see Supplementary Table S2, Supplemental digital content 2 http://links.lww.com/MR/A259 that presents the patient characteristics). The median distant metastasis-free interval (DMFI) was 26* *months (range, 0–265; IQR 13–54; see Supplementary Figure S2, Supplemental digital content 1, http://links.lww.com/MR/A258).

Ninety-two percent of patients had hepatic metastases with or without dissemination to other sites, and 70% had only hepatic metastases (see Supplementary Table S2, Supplemental digital content 2, http://links.lww.com/MR/A259). The median LDLM was 30 mm (range, 2–196). The largest metastasis was small (M1a) in 52%, medium-sized (M1b) in 31%, large (M1c) in 11%, and undetermined in 6% of patients. Serum or plasma alkaline phosphatase exceeded the upper normal limit (UNL) in 26% of 193 patients with available data. Performance status was 0–2 for 96% and 3–4 for 4% of patients, of whom 56 and 43% represented M1a, and 30 and 67% had elevated alkaline phosphatase levels, respectively. Of 213 successfully staged patients, 143 (67%) fell in the Helsinki University Hospital Working Formulation stage IVa, 37 (17%) in IVb, and 33 (15%) in IVc.

At treatment decision, the median age was 64* *years (range, 21–86) and the median interval from diagnosis of metastases 56 days (range, 0–1059; IQR, 34–92; see Supplementary Table S3, Supplemental digital content 2, http://links.lww.com/MR/A259 that reports reasons for delays exceeding 90 days in 59 patients).

Of the 216 patients, 104 (48%) received first-line chemoimmunotherapy and 43 (20%) conventional chemotherapy, 19 (9%) underwent surgery and 22 (10%) SIRT, 14 (6%) received IFN-alpha or IL-2 monotherapy and 8 (4%) a CPI, 3 (1%) underwent TACE and 2 (1%) LDT, and one received a PKI (Table 1; Supplementary Figure S3A, Supplemental digital content 1, http://links.lww.com/MR/A258). The majority of patients who received chemoimmunotherapy were given IFN-alpha with bleomycin, vincristine, lomustine and dacarbazine (46%) [29–31] or with dacarbazine alone (43%). Forty-three percent of patients received more than one line of treatment (median, 3; Supplementary Figure S3B, Supplemental digital content 1, http://links.lww.com/MR/A258). Comparison between second-line therapies was not possible because of less than 10 patients in all subgroups. Twelve (6%) patients participated in four treatment trials (NCT02599402, NCT01974752, NCT00154388 and NCT00308607).

**Table 1:** Deaths, overall survival, Helsinki University Hospital Working Formulation stages, and stage-specific overall survival, and comparison against best supportive care by treatment modality

Treatment	Deaths/all patients, *n*	Median overall survival, months (95% CI)	Working formulation stage IVa/IVb/IVc, *n (%*)	Median overall survival by stage, months (95% CI)			Overall survival compared to BSC	
				IVa	IVb	IVc	Hazard ratio (95% CI)	*P* value
Systemic	166/170	11 (8.7–13)	103 (62)/32 (19)/32 (19)	16 (13–19)	6.0 (4.6–9.6)	1.9 (1.6–3.0)	1.92 (1.50–2.45)	<0.001
CHT	43/43	5.1 (3.0–8.0)	19 (44)/9 (21)/15 (35)	10 (4.8–14)	6.9 (1.3–12)	2.4 (1.0–3.3)	1.29 (0.90–1.84)	0.17
CIT	103/104	13 (10–16)	70 (69)/17 (17)/14 (14)	18 (15–21)	6.0 (4.5–9.6)	1.9 (1.5–5.2)	2.10 (1.60–2.77)	<0.001
IFN/IL	14/14	9.0 (2.9–18)	8 (57)/3 (21)/3 (21)	14 (9.0–31)	4.6 (3.4–N/A)	1.5 (0.7–N/A)	1.60 (0.92–2.81)	0.096
CPI	5/8	13 (4.0–N/A)	5 (63)/3 (37)/0 (0)	N/A^a^	12 (4.1–N/A)	N/A	3.30 (1.34–8.13)	0.010
PKI	1/1	N/A	1 (100)/0 (0)/0 (0)	9.7^b^	N/A	N/A	1.46 (0.20–10.5)	0.71
Local	36/46	23 (16–30)	40 (87)/5 (11)/1 (2)	25 (17–40)	9.7 (2.9–N/A)	0.3^b^	3.49 (2.35–5.17)	<0.001
Surgery	15/19	24 (16–73)	17 (89)/1 (5)/1 (5)	27 (17–73)	7.2^b^	0.3^b^	3.88 (2.19–6.88)	<0.001
SIRT	17/22	16 (9.0–30)	19 (86)/3 (14)/0 (0)	24 (9.0–30)	9.7 (2.9–N/A)	N/A	2.76 (1.65–4.64)	<0.001
TACE	3/3	16 (12–N/A)	2 (67)/1 (33)/0 (0)	16; 41^b^	12^b^	N/A	2.39 (0.75–7.57)	0.14
LDT	1/2	73 (N/A)	2 (100)/0 (0)/0 (0)	31; 73^b^	N/A	N/A	7.53 (1.03–55.1)	0.047
BSC	107/108	1.6 (0.9–2.9)	26 (24)/20 (19)/59 (55)	12 (9.4–21)	5.7 (0.7–11)	0.6 (0.3–0.9)		

BSC, best supportive care; CHT, conventional chemotherapy; CI, confidence interval; CIT, chemoimmunotherapy with interferon or interleukin; CPI, checkpoint inhibitor; IFN/IL, interferon-alpha or interleukin-2 monotherapy; LDT, other liver-directed therapies; N/A, not applicable; PKI, protein kinase inhibitor; SIRT, selective internal radiation therapy; TACE, trans-arterial chemoembolization.

a Median not reached.

b Median not calculable, individual survival given.

### Stage-specific overall survival

Of the 216 patients, 14 were alive with metastases at the time of analysis. The audited primary cause of death was metastatic uveal melanoma for all others. The median OS across all treatment modalities was 12 months (95% CI, 11–14; range, 0.2–162; Supplementary Figure S4A, Supplemental digital content 1, http://links.lww.com/MR/A258). Survival shortened with increasing working formulation stage from 18 (range, 0.7–162) to 6.9 (range, 1.3–30) and 1.9 (range, 0.2–18) months for stage IVa, IVb and IVc, respectively (*P* < 0.001, log-rank test for trend, Supplementary Figure S4B, S4C and S4D, Supplemental digital content 1, http://links.lww.com/MR/A258 show stage-specific OS with systemic and local therapy, respectively). In stage IVa, 73% of patients survived at least 12 months from the treatment decision, in stage IVb, 57 and 19% survived at least 6 and 12 months, respectively, and in stage IVc, 88% died within 6 months. The weighted kappa for agreement between observed and predicted OS category was 0.549 (agreement 81 vs. 58% expected, *P* < 0.001, Supplementary Table S4, Supplemental digital content 2, http://links.lww.com/MR/A259; Supplementary Figure S5, Supplemental digital content 1, http://links.lww.com/MR/A258), calculated from the treatment decision [17].

**Fig. 1:**
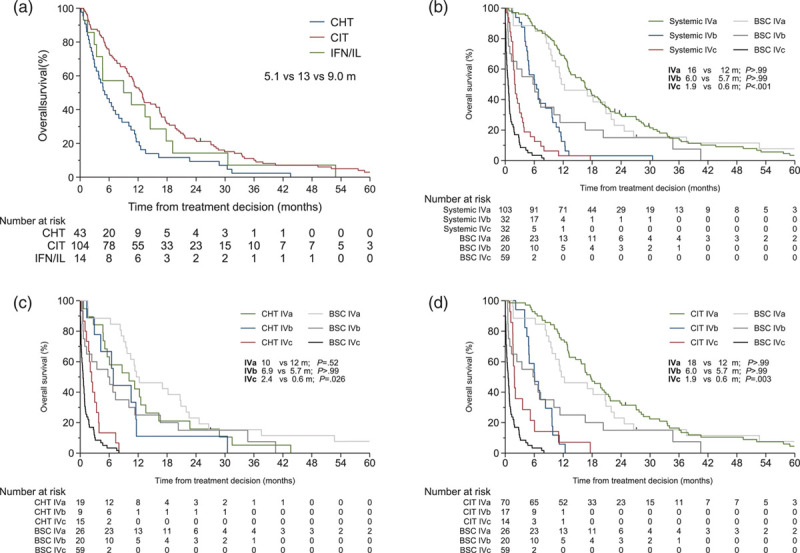
Kaplan–Meier graph of overall survival (OS) from first-line treatment decision. (a) By systemic treatments, shown are treatment modalities with >10 patients, and for (b) any systemic treatment, (c) conventional chemotherapy (CHT) and (d) chemoimmunotherapy with interferon or interleukin (CIT) against best supportive care (BSC) by the Helsinki University Hospital Working Formulation stage. Median OS and *P* value are given, calculated by the log-rank test, with Bonferroni correction in B-D. For other abbreviations, see Table 1.

Regarding the three systemic treatment modalities given to at least 10 patients, the median OS was longest, 13 months, with chemoimmunotherapy as compared with conventional chemotherapy and IFN/IL monotherapy (5 and 9 months, respectively; Fig. 1a), but the longer OS with chemoimmunotherapy was restricted to stage IVa (Table 1; *P* = 0.013 compared to conventional chemotherapy, log-rank test with Bonferroni correction for three comparisons). OS with various chemoimmunotherapy regimens was comparable, regardless of working formulation stage (Supplementary Figure S6A, Supplemental digital content 1, http://links.lww.com/MR/A258). OS after systemic therapy (Fig. 1b), conventional chemotherapy (Fig. 1c) and chemoimmunotherapy (Fig. 1d) did not differ from that with BSC in stage IVa and IVb, although it was 1–2 months longer in stage IVc (*P* < 0.001, *P* = 0.026 and *P* = 0.003 for systemic therapy, conventional chemotherapy and chemoimmunotherapy, respectively). OS with IFN/IL monotherapy did not differ from that with BSC (Supplementary Figure S6B, Supplemental digital content 1, http://links.lww.com/MR/A258), and too few patients received CPI to confirm longer OS than with BSC (Table 1; Supplementary Figure S6C, Supplemental digital content 1, http://links.lww.com/MR/A258).

**Fig. 2:**
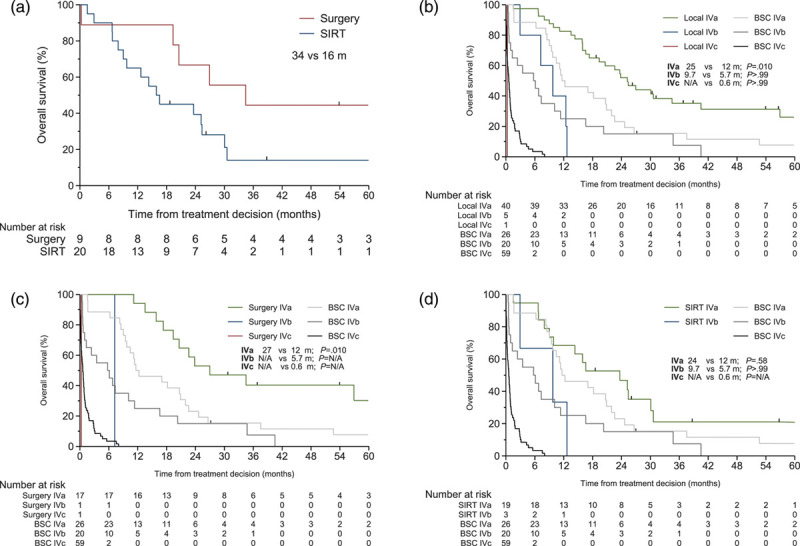
Kaplan–Meier graph of overall survival (OS) from first-line treatment decision. (a) By local treatments, shown are treatment modalities with >10 patients and of those patients with only hepatic metastases, and for (b) any local treatment, (c) surgery and (d) selective internal radiation therapy (SIRT) against best supportive care (BSC) by the Helsinki University Hospital Working Formulation stage. Median OS and *P* value are given, calculated by the log-rank test, with Bonferroni correction in B-D. For other abbreviations, see Table 1.

Considering the two local treatments given to at least 10 patients, the median OS was longer after surgical resection of hepatic metastases, 34 months, than with SIRT (16 months, *P* = 0.002; Fig. 2a; for the type of surgical intervention, see Table S5, Supplemental digital content 2, http://links.lww.com/MR/A259). OS after local treatment (Fig. 2b) and surgery (Fig. 2c) was longer than with BSC in working formulation stage IVa (*P* = 0.010 and *P* = 0.010, respectively, log-rank test with Bonferroni correction), but with SIRT it was comparable to that with BSC (*P* = 0.58; Fig. 2d). Not enough patients in stage IVb and IVc were treated to allow comparison with BSC for any local therapy. When considering patients with only hepatic metastases, no difference in OS was observed between chemoimmunotherapy and SIRT either (*P* > 0.99; Supplementary Figure S7, Supplemental digital content 1, http://links.lww.com/MR/A258; Supplementary Table S6, Supplemental digital content 2, http://links.lww.com/MR/A259).

### Predictors of overall survival

By univariable Cox regression, working formulation stage predicted OS as expected [17] (Supplementary Table S7, Supplemental digital content 2, http://links.lww.com/MR/A259). Regarding the components of working formulation (Supplementary Figure S8, Supplemental digital content 1, http://links.lww.com/MR/A258), median OS was 14 (range, 13–17), 3.8 (range, 1.5–5.0) and 2.9 (range, 0.7–3.9) months for performance status 0–1, 2 and 3–4, respectively. A higher alkaline phosphatase and larger LDLM were also associated with shorter OS.

Working formulation stages were not entirely homogenous regarding these predictors (Supplementary Figure S8, Supplemental digital content 1, http://links.lww.com/MR/A258). In stage IVa, only three patients had performance status >1 and only two an alkaline phosphatase level >2.0 × UNL, but 29% had an LDLM >M1a with shorter OS (*P* = 0.018, log-rank test). In stage IVb, 27% of patients had performance status >1 and possibly shorter OS (*P* = 0.018), whereas alkaline phosphatase and LDLM contributed no significant heterogeneity. In stage IVc, performance status and LDLM did not contribute to heterogeneity, and only four patients had a more favorable alkaline phosphatase level <1.0 × UNL.

Analyzed by working formulation stage (Supplementary Figure S9, Supplemental digital content 1, http://links.lww.com/MR/A258), lactate dehydrogenase (LDH) >2.0 × UNL was associated with shorter OS in stage IVa (*P* = 0.002; log-rank test for trend), whereas gender, age, presence of symptoms and sites of metastases were unassociated with OS in any stage. Also, DMFI was associated with OS in stage IVa revealing a dichotomy in which OS was longer if DMFI exceeded 3.5 years (*P* < 0.001).

In bivariable models including working formulation stage, only LDH >2.0 × UNL was independently associated with OS (*P* = 0.002, HR 4.76; Supplementary Table S7, Supplemental digital content 2, http://links.lww.com/MR/A259) and this model fitted better with data than working formulation stage alone (−2 log likelihood = 427.89 vs. 734.35, *P* < 0.001, *df* = 2). Adding also symptoms from metastases did not improve the model (−2 log likelihood = 427.89 vs. 427.78, *P* = 0.79, *df* = 2). Combining working formulation stage either with presence of symptoms or DMFI, which has earlier been proposed as an independent predictor [32–34], did not improve the model (*P* = 0.44 and *P* = 0.63, respectively, deviance test).

## Discussion

Our stage-specific, real-life, nationwide OS data of actively treated metastatic uveal melanoma suggest that OS with all treatment modalities administered to at least 10 patients was comparable to that with our BSC cohort [18], except for patients who underwent surgical resection of metastases representing the most favourable Helsinki University Hospital Working Formulation stage IVa. Although OS after systemic chemoimmunotherapy and local SIRT exceeded that with conventional chemotherapy, it was comparable to that with BSC, except in working formulation stage IVc. However, patients receiving BSC in stage IVc had worse performance status, which likely explains the difference [18]. We had too few patients to draw conclusions about CPIs. Our results suggest that BSC rather than conventional chemotherapy may be the best reference against which to compare a novel treatment in a retrospective setting, unlike repeatedly has been done [8,9,13,35,36]. Adding a historical BSC benchmark [18] might enhance even the analysis of a prospective trial.

Especially local treatments have been suggested to prolong survival, based on median OS of 18–35 months [3–6]. We confirmed longer OS by surgical resection as compared to BSC in working formulation stage IVa. In Finland, SIRT has been used since 2010 as first-line local treatment for metastatic uveal melanoma restricted to the liver when surgical resection is unfeasible. Of note, only 9% of our patients received local treatment as compared to 22% in an earlier nationwide Dutch series [14]. Our results suggest that SIRT is not superior to previously preferred chemoimmunotherapy or, indeed, to BSC, considering stage-specific OS.

The working formulation stages in our actively treated cohort were skewed towards IVa as compared to previous studies [24,29,30,37]. The OS in stage IVa differed even more from IVb than it did in the building and validation datasets [17,24], in part because survival in stage IVb was shorter than in the earlier data. This may reflect more active follow-up strategies leading to earlier detection of metastases in our cohort, resulting in stage migration and shortened DMFI.

Although working formulation staging divided patients in three groups with clearly different OS, and the three components of working formulation were strongly associated with OS, the working formulation stages were not entirely homogenous with regard to these predictors. No consistent source of bias over all stages was detected in these components, however. LDH has previously been proposed to be an independent prognostic factor in patients with metastatic uveal melanoma [32,34,38]. Although LDH was available only for a subpopulation of patients, our data support that working formulation staging might benefit from additionally considering LDH. We confirmed that DMFI >3.5 years, another proposed independent predictor of OS [32,34,38], was associated with longer OS on univariable level; however, it did not improve working formulation staging.

The main limitations of our study are its retrospective nature and case-wise selection of patients for treatments by preference of the managing oncologist and the patient. Recent real-life studies from tertiary centers [4,6,15,16] and the nationwide Dutch study [14] had gaps in design, data collection, and analysis: data regarding specific treatments was only available for 30% of patients in one cohort [15], and in the nationwide study performance status was unavailable for 36% of those who received local treatment [14]. None of the studies staged the patients. In our cohort, specific treatment was always known, performance status was missing from one patient, 99% were staged using a validated system [17], and an identically staged, concurrent cohort receiving BSC was available for comparison [18]. Our cohort also has similarities with the previous real-life studies: the DMFI, sites of metastases, LDLM, alkaline phosphatase levels and performance status were similar to those that they reported [6,14,15,24].

All patients diagnosed with metastatic uveal melanoma limited to the liver are discussed in our multidisciplinary meeting. Given the present results, we strive to offer surgical resection for all eligible patients [33,39]. Other patients with localized hepatic metastases are considered for SIRT. We have lowered the threshold to recommend BSC for patients who are elderly or have a reduced performance status [18] but attempt to enroll patients whose performance status is favourable to the ongoing trials in which our center participates (currently NCT03733990) [40,41]. We advocate systematic collection of biopsies from metastases to promote the discovery of any other subcategories amenable to specific treatments such as CPI for patients who carry a pathogenic variant in *MBD4* [42,43] though such subcategories may be small [44,45]. The inclusion of patients with metastatic uveal melanoma in clinical trials is crucial to eventually benefit the majority of patients.

### Conclusion

Our stage-specific, real-life, nationwide outcome data of actively treated metastatic uveal melanoma suggest that no treatment available to most patients appreciably prolonged OS. Surgical resection may have been beneficial in stage IVa, but few were eligible. A better reference against which to compare OS in retrospective studies might be a BSC benchmark such as ours, available at *https://doi.org/10.5281/zenodo.3369090*, rather than conventional chemotherapy. Especially, our data highlight the importance of staging of patients with metastases when comparing survival outcomes.

## Acknowledgements

The authors thank all treating physicians. Data and specimens were obtained with permission from Helsinki Biobank, Biobank Borealis, Biobank of Eastern Finland; Fimlab Laboratories; University Hospitals of Kuopio, Tampere, Turku and Oulu; sixteen Central Hospitals; private health care providers Terveystalo, Pihlajalinna Koskiklinikka, and Docrates Cancer Centre; several district hospitals; and many health care centers. Technical assistance of Ms. Seija Lehtonen is gratefully acknowledged.

E.S.R. received grants from Mary and Georg C. Ehrnrooth Foundation, Eye Foundation, Eye and Tissue Bank Foundation, Evald and Hilda Nissi Foundation, Finnish Medical Foundation, and Helsinki University Hospital Research Fund; all in Finland. T.T.K. received grants from Finnish Cancer Foundation, Sigrid Jusélius Foundation, and Helsinki University Hospital Research Fund (grant nos. TYH2017218 and TYH2020315); all in Finland. The funding source had no influence on design, collection, analysis and writing of the article.

E.S.R. conceptualized and designed the study, responsible for data-analysis decisions, management and retrieval of data, decided on and contributed to data-collection methods and initial data analysis and interpretation, drafted the initial article, and approved the final version. T.T.K. conceptualized and designed the study, responsible for data-analysis decisions, management and retrieval of data, decided on and contributed to data-collection methods and initial data analysis and interpretation, critically reviewed and revised the article, supervised all aspects of the study, and approved the final version. M.M.H. conceptualized and designed the study, responsible for data-analysis decisions, contributed to initial data analysis and interpretation, critically reviewed and revised the article, supervised all aspects of the study, and approved the final version. All authors meet the International Committee of Medical Journal Editors criteria for authorship.

## Conflicts of interest

E.S.R. reports personal fees from Théa Nordic. M.M.H. reports personal fees from BMS, MSD, Novartis, Roche, Sanofi, and Varian. T.T.K. reports personal fees from Santen Finland; all outside the submitted work.

## Supplementary Material



## References

[1] Kujala E Mäkitie T Kivelä T. Very long-term prognosis of patients with malignant uveal melanoma. Invest Ophthalmol Vis Sci. 2003; 44:4651–4659. 14578381 10.1167/iovs.03-0538

[2] Singh AD Turell ME Topham AK. Uveal melanoma: trends in incidence, treatment, and survival. Ophthalmology. 2011; 118:1881–1885. 21704381 10.1016/j.ophtha.2011.01.040

[3] Moser JC Pulido JS Dronca RS McWilliams RR Markovic SN Mansfield AS. The Mayo Clinic experience with the use of kinase inhibitors, ipilimumab, bevacizumab, and local therapies in the treatment of metastatic uveal melanoma. Melanoma Res. 2015; 25:59–63. 25396683 10.1097/CMR.0000000000000125

[4] Xu LT Funchain PF Bena JF Li M Tarhini A Berber E Singh AD. Uveal melanoma metastatic to the liver: treatment trends and outcomes. Ocul Oncol Pathol. 2019; 5:323–332. 31559243 10.1159/000495113 PMC6751472

[5] Akyuz M Yazici P Dural C Yigitbas H Okoh A Bucak E. Laparoscopic management of liver metastases from uveal melanoma. Surg Endosc. 2016; 30:2567–2571. 26310535 10.1007/s00464-015-4527-9

[6] Seedor RS Eschelman DJ Gonsalves CF Adamo RD Orloff M Amjad A. An outcome assessment of a single institution’s longitudinal experience with uveal melanoma patients with liver metastasis. Cancers (Basel). 2020; 12:117. 10.3390/cancers12010117 PMC7016993 31906411

[7] Rantala ES Hernberg M Kivelä TT. Overall survival after treatment for metastatic uveal melanoma: a systematic review and meta-analysis. Melanoma Res. 2019; 29:561–568. 30664106 10.1097/CMR.0000000000000575 PMC6887637

[8] Leyvraz S Piperno-Neumann S Suciu S Baurain JF Zdzienicki M Testori A. Hepatic intra-arterial versus intravenous fotemustine in patients with liver metastases from uveal melanoma (EORTC 18021): a multicentric randomized trial. Ann Oncol. 2014; 25:742–746. 24510314 10.1093/annonc/mdt585 PMC4433517

[9] Carvajal RD Piperno-Neumann S Kapiteijn E Chapman PB Frank S Joshua AM. Selumetinib in combination with dacarbazine in patients with metastatic uveal melanoma: a phase III, multicenter, randomized trial (SUMIT). J Clin Oncol. 2018; 36:1232–1239. 29528792 10.1200/JCO.2017.74.1090

[10] Valsecchi ME Terai M Eschelman DJ Gonsalves CF Chervoneva I Shields JA. Double-blinded, randomized phase II study using embolization with or without granulocyte-macrophage colony-stimulating factor in uveal melanoma with hepatic metastases. J Vasc Interv Radiol. 2015; 26:523–32.e2. 25678394 10.1016/j.jvir.2014.11.037 PMC4417549

[11] Agarwala SS Panikkar R Kirkwood JM. Phase I/II randomized trial of intrahepatic arterial infusion chemotherapy with cisplatin and chemoembolization with cisplatin and polyvinyl sponge in patients with ocular melanoma metastatic to the liver. Melanoma Res. 2004; 14:217–222. 15179192 10.1097/01.cmr.0000129377.22141.ea

[12] Daud A Kluger HM Kurzrock R Schimmoller F Weitzman AL Samuel TA. Phase II randomised discontinuation trial of the MET/VEGF receptor inhibitor cabozantinib in metastatic melanoma. Br J Cancer. 2017; 116:432–440. 28103611 10.1038/bjc.2016.419 PMC5318966

[13] Luke JJ Olson DJ Allred JB Strand CA Bao R Zha Y. Randomized phase II trial and tumor mutational spectrum analysis from cabozantinib versus chemotherapy in metastatic uveal melanoma (alliance A091201). Clin Cancer Res. 2020; 26:804–811. 31558480 10.1158/1078-0432.CCR-19-1223 PMC7055933

[14] Jochems A van der Kooij MK Fiocco M Schouwenburg MG Aarts MJ van Akkooi AC. Metastatic uveal melanoma: treatment strategies and survival-results from the Dutch melanoma treatment registry. Cancers (Basel). 2019; 11:1007. 10.3390/cancers11071007 PMC6678641 31323802

[15] Lane AM Kim IK Gragoudas ES. Survival rates in patients after treatment for metastasis from uveal melanoma. JAMA Ophthalmol. 2018; 136:981–986. 29955797 10.1001/jamaophthalmol.2018.2466 PMC6142974

[16] Nicholas MN Khoja L Atenafu EG Hogg D Quirt I Butler M Joshua AM. Prognostic factors for first-line therapy and overall survival of metastatic uveal melanoma: the Princess Margaret Cancer Centre experience. Melanoma Res. 2018; 28:571–577. 30067547 10.1097/CMR.0000000000000468

[17] Kivelä TT Piperno-Neumann S Desjardins L Schmittel A Bechrakis N Midena E. Validation of a prognostic staging for metastatic uveal melanoma: a collaborative study of the European Ophthalmic Oncology Group. Am J Ophthalmol. 2016; 168:217–226. 27296487 10.1016/j.ajo.2016.06.002

[18] Rantala ES Hernberg M Lundin M Lundin J Kivelä TT. Metastatic uveal melanoma managed with best supportive care. Acta Oncol. 2021; 60:135–139. 32960119 10.1080/0284186X.2020.1817978

[19] Kivelä TT Simpson ER Grossniklaus HE Jager MJ Singh AD Caminal JM. Uveal melanoma. Amin MB Edge S Greene F Byrd DR Brookland RK Washington MK, editors. In: AJCC cancer staging manual. 8th ed. Chicago, USA: Springer International Publishing; 2017. pp. 813–826.

[20] Kujala E Damato B Coupland SE Desjardins L Bechrakis NE Grange JD Kivelä T. Staging of ciliary body and choroidal melanomas based on anatomic extent. J Clin Oncol. 2013; 31:2825–2831. 23816968 10.1200/JCO.2012.45.2771

[21] Eskelin S Pyrhönen S Summanen P Prause JU Kivelä T. Screening for metastatic malignant melanoma of the uvea revisited. Cancer. 1999; 85:1151–1159. 10091801

[22] Oken MM Creech RH Tormey DC Horton J Davis TE McFadden ET Carbone PP. Toxicity and response criteria of the Eastern Cooperative Oncology Group. Am J Clin Oncol. 1982; 5:649–655. 7165009

[23] Moy CS Albert DM Diener-West M McCaffrey LD Scully RE Willson JK; Collaborative Ocular Melanoma Study Group, prepared by COMS Mortality Coding Committee. Cause-specific mortality coding. Methods in the collaborative ocular melanoma study COMS report no. 14. Control Clin Trials. 2001; 22:248–262. 11384789 10.1016/s0197-2456(01)00113-1

[24] Eskelin S Pyrhönen S Hahka-Kemppinen M Tuomaala S Kivelä T. A prognostic model and staging for metastatic uveal melanoma. Cancer. 2003; 97:465–475. 12518371 10.1002/cncr.11113

[25] US Food and Drug Administration. Clinical trial endpoints for the approval of cancer drugs and biologics - guidance for industry. 2018; https://www.fda.gov/media/71195/download. [Accessed 6 July 2020].

[26] European Medicines Agency. Guideline on the evaluation of anticancer medicinal products in man. 2005; https://www.ema.europa.eu/en/documents/scientific-guideline/guideline-evaluation-anticancer-medicinal-products-man-revision-3_en.pdf. [Accessed 6 July 2020].

[27] Peduzzi P Concato J Feinstein AR Holford TR. Importance of events per independent variable in proportional hazards regression analysis. II. Accuracy and precision of regression estimates. J Clin Epidemiol. 1995; 48:1503–1510. 8543964 10.1016/0895-4356(95)00048-8

[28] Therneau TM Grambsch PM. Modeling survival data: extending the Cox model. New York, USA: Springer-Verlag; 2000.

[29] Pyrhönen S Hahka-Kemppinen M Muhonen T Nikkanen V Eskelin S Summanen P. Chemoimmunotherapy with bleomycin, vincristine, lomustine, dacarbazine (BOLD), and human leukocyte interferon for metastatic uveal melanoma. Cancer. 2002; 95:2366–2372. 12436444 10.1002/cncr.10996

[30] Kivelä T Suciu S Hansson J Kruit WH Vuoristo MS Kloke O. Bleomycin, vincristine, lomustine and dacarbazine (BOLD) in combination with recombinant interferon alpha-2b for metastatic uveal melanoma. Eur J Cancer. 2003; 39:1115–1120. 12736111 10.1016/s0959-8049(03)00132-1

[31] Nathan FE Berd D Sato T Shield JA Shields CL De Potter P Mastrangelo MJ. BOLD+interferon in the treatment of metastatic uveal melanoma: first report of active systemic therapy. J Exp Clin Cancer Res. 1997; 16:201–208. 9261748

[32] Mariani P Dureau S Savignoni A Rouic LL Levy-Gabriel C Piperno-Neumann S. Development of a prognostic nomogram for liver metastasis of uveal melanoma patients selected by liver MRI. Cancers (Basel). 2019; 11:863. 10.3390/cancers11060863 PMC6627813 31234340

[33] Mariani P Piperno-Neumann S Servois V Berry MG Dorval T Plancher C. Surgical management of liver metastases from uveal melanoma: 16 years’ experience at the Institut Curie. Eur J Surg Oncol. 2009; 35:1192–1197. 19329272 10.1016/j.ejso.2009.02.016

[34] Valpione S Moser JC Parrozzani R Bazzi M Mansfield AS Mocellin S. Development and external validation of a prognostic nomogram for metastatic uveal melanoma. PLoS One. 2015; 10:e0120181. 25780931 10.1371/journal.pone.0120181 PMC4363319

[35] Tulokas S Mäenpää H Peltola E Kivelä T Vihinen P Virta A. Selective internal radiation therapy (SIRT) as treatment for hepatic metastases of uveal melanoma: a Finnish nation-wide retrospective experience. Acta Oncol. 2018; 57:1373–1380. 29683787 10.1080/0284186X.2018.1465587

[36] Carling U Dorenberg EJ Haugvik SP Eide NA Berntzen DT Edwin B. Transarterial chemoembolization of liver metastases from uveal melanoma using irinotecan-loaded beads: treatment response and complications. Cardiovasc Intervent Radiol. 2015; 38:1532–1541. 25832764 10.1007/s00270-015-1093-4

[37] Vihinen PP Hernberg M Vuoristo MS Tyynelä K Laukka M Lundin J. A phase II trial of bevacizumab with dacarbazine and daily low-dose interferon-alpha2a as first line treatment in metastatic melanoma. Melanoma Res. 2010; 20:318–325. 20375744 10.1097/CMR.0b013e3283390365

[38] Lorenzo D Ochoa M Piulats JM Gutiérrez C Arias L Català J. Prognostic factors and decision tree for long-term survival in metastatic uveal melanoma. Cancer Res Treat. 2018; 50:1130–1139. 29198096 10.4143/crt.2017.171 PMC6192913

[39] Gomez D Wetherill C Cheong J Jones L Marshall E Damato B. The Liverpool uveal melanoma liver metastases pathway: outcome following liver resection. J Surg Oncol. 2014; 109:542–547. 24357463 10.1002/jso.23535

[40] Rao PK Barker C Coit DG Joseph RW Materin M Rengan R; ScM. NCCN guidelines insights: uveal melanoma, version 1.2019. J Natl Compr Canc Netw. 2020; 18:120–131. 32023525 10.6004/jnccn.2020.0007

[41] Seth R Messersmith H Kaur V Kirkwood JM Kudchadkar R McQuade JL. Systemic therapy for melanoma: ASCO guideline. J Clin Oncol. 2020; 38:3947–3970. 32228358 10.1200/JCO.20.00198

[42] Rodrigues M Mobuchon L Houy A Fiévet A Gardrat S Barnhill RL. Outlier response to anti-PD1 in uveal melanoma reveals germline MBD4 mutations in hypermutated tumors. Nat Commun. 2018; 9:1866. 29760383 10.1038/s41467-018-04322-5 PMC5951831

[43] Johansson PA Stark A Palmer JM Bigby K Brooks K Rolfe O. Prolonged stable disease in a uveal melanoma patient with germline MBD4 nonsense mutation treated with pembrolizumab and ipilimumab. Immunogenetics. 2019; 71:433–436. 30714079 10.1007/s00251-019-01108-x

[44] Derrien AC Rodrigues M Eeckhoutte A Dayot S Houy A Mobuchon L. Germline MBD4 mutations and predisposition to uveal melanoma. J Natl Cancer Inst. 2021; 113:80–87. 32239153 10.1093/jnci/djaa047 PMC7781447

[45] Repo P Jäntti JE Järvinen RS Rantala ES Täll M Raivio V. Germline loss-of-function variants in MBD4 are rare in Finnish patients with uveal melanoma. Pigment Cell Melanoma Res. 2020; 33:756–762. 32421892 10.1111/pcmr.12892

