# Supplementary material for: Impact of staging on survival outcomes: a nationwide real-world cohort study of metastatic uveal melanoma
Source: Melanoma Res. 2021 Mar 5;31(3):224–31. doi: 10.1097/CMR.0000000000000728 (PMC8081447; doi:10.1097/CMR.0000000000000728)
Supplement: Supplementary file 1 [file mr-31-224-s001.pdf]

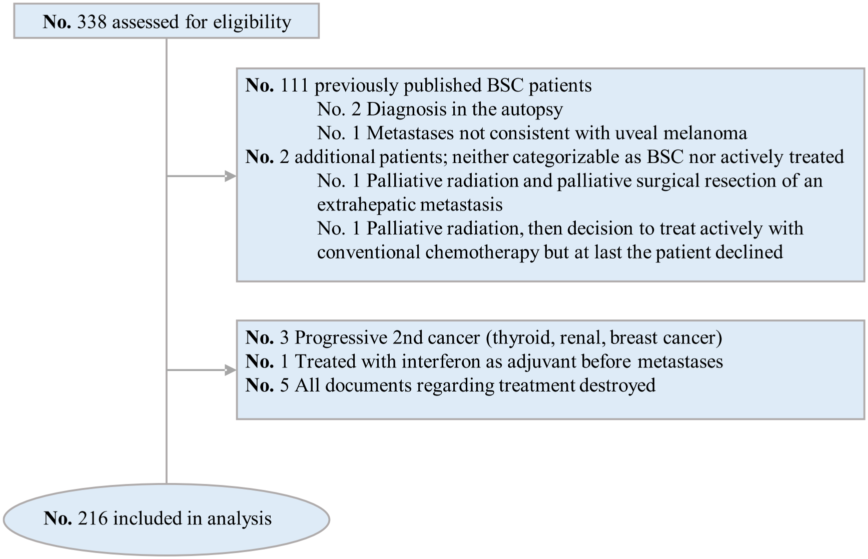

Fig. S1. Study flow chart. No. 216 included in analysis. BSC, best supportive care.

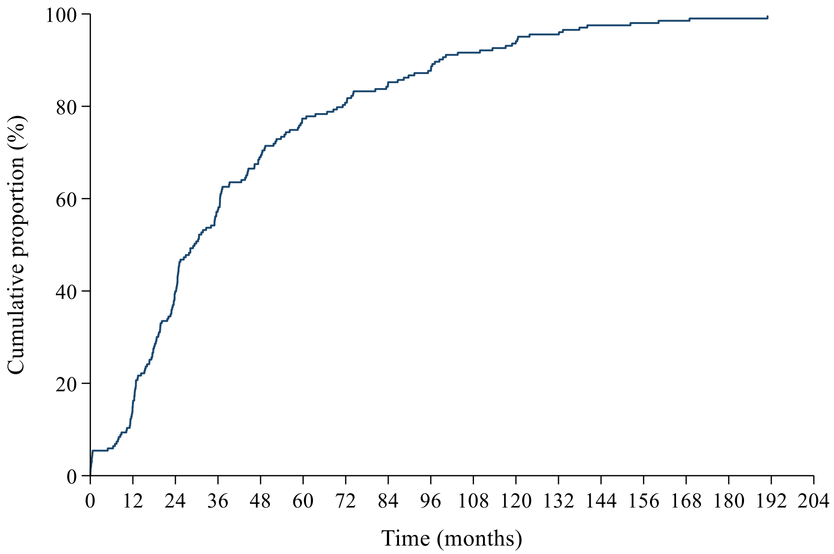

Fig. S2. Cumulative frequency plot of distant metastasis-free interval to the diagnosis of metastases.

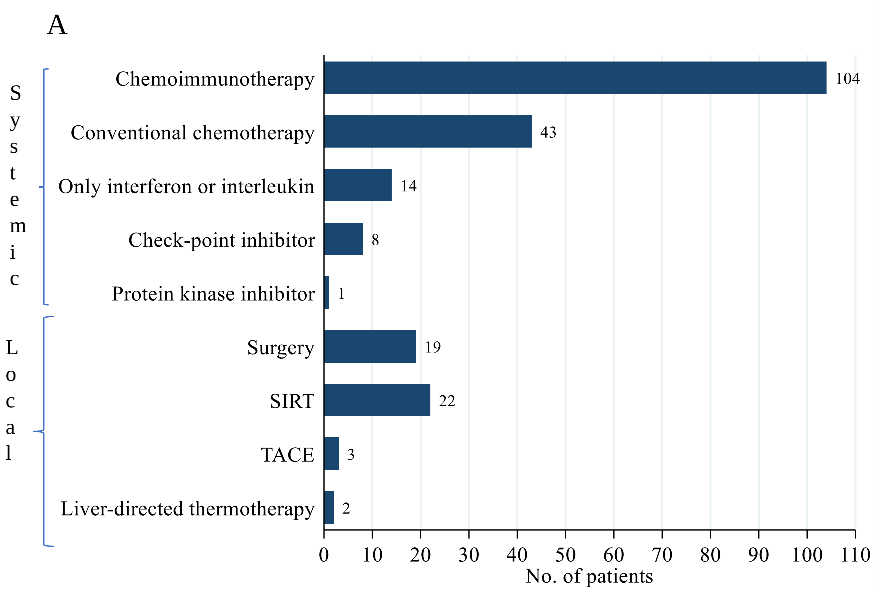

Fig. S3. Treatment modalities. (A) 1st-line treatment modalities and number of patients; (B) Treatment lines and treatment modalities for the first five treatment lines. The median time between the initiation of the 1st- and 2nd-line treatment was 129 days (interquartile range, 88-204). CHT, conventional chemotherapy; CIT, chemoimmunotherapy with IFN/IL; CPI, check-point inhibitor; IFN/IL, interferon or interleukin; LDT, other liver-directed therapies; PKI, protein kinase inhibitor; SIRT, selective internal radiation therapy; TACE, trans-arterial chemoembolization.

B

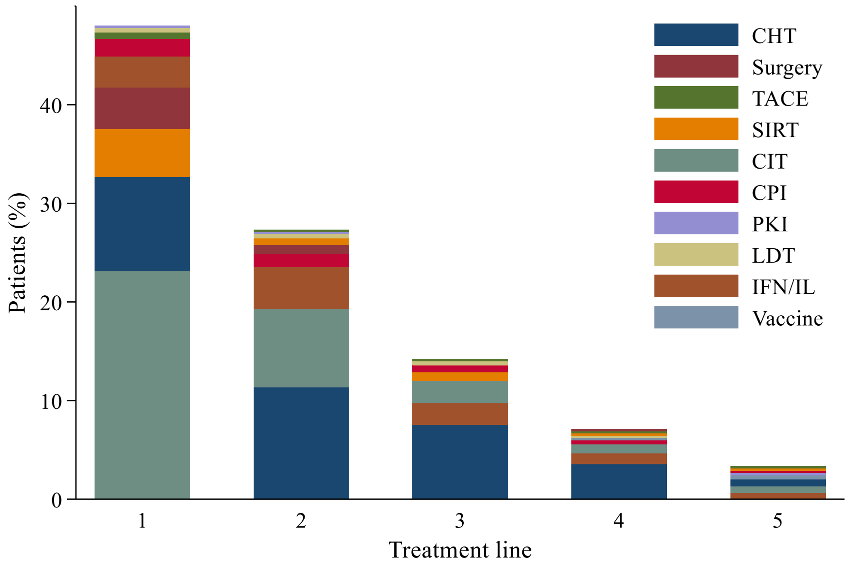
CHT
Surgery
TACE
SIRT
CIT
CPI
PKI
LDT
IFN/IL
Vaccine
Patients (%)
0
10
20
30
40
1
2
3
4
5
Treatment line

A

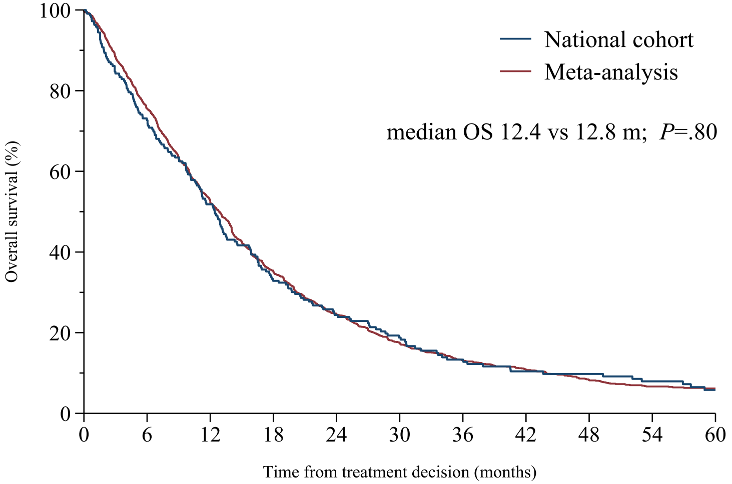

Number at risk

	0	6	12	18	24	30	36	42	48	54	60
National cohort	216	157	112	71	50	36	24	17	16	12	8
Meta-analysis	2494	1808	1184	729	470	296	197	144	96	65	35

Fig. S4. Kaplan-Meier graph of overall survival (OS) from decision to treat to death, shaded area shows the confidence intervals. (A) Entire actively treated cohort against the meta-analysis of 2,494 patients [Rantala et al. Overall survival after treatment for metastatic uveal melanoma: a systematic review and meta-analysis. Melanoma Res. 2019 Dec;29(6):561-568].; (B) according to the Helsinki University Hospital Working Formulation (WF), three patients could not be staged and were omitted; (C) systemic treatment modalities according to the WF; (D) local therapy according to the WF.

B

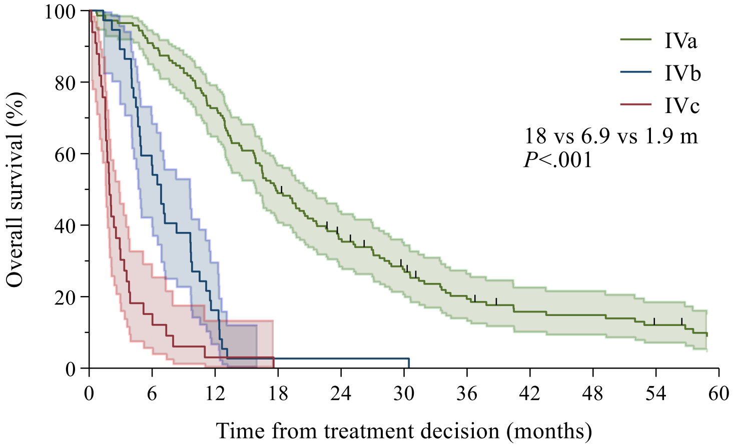

Number at risk

IVa	143	130	104	70	49	35	23	17	16	12	8
IVb	37	21	6	1	1	1	0	0	0	0	0
IVc	33	5	1	0	0	0	0	0	0	0	0

C

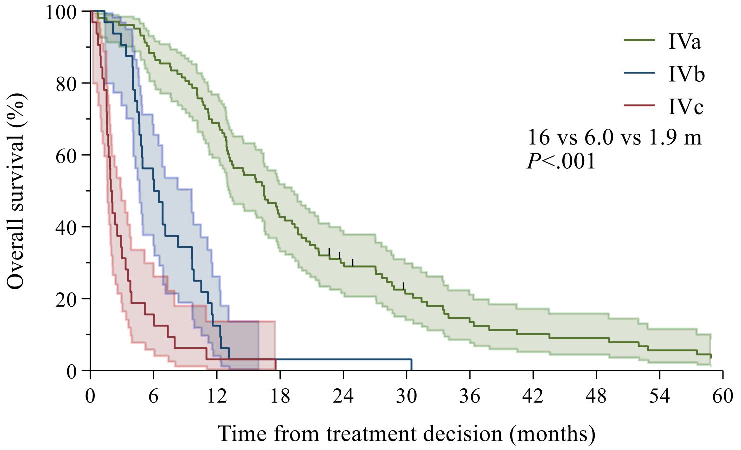

Number at risk

IVa	103	91	71	44	29	19	12	9	8	5	3
IVb	32	17	4	1	1	1	0	0	0	0	0
IVc	32	5	1	0	0	0	0	0	0	0	0

D

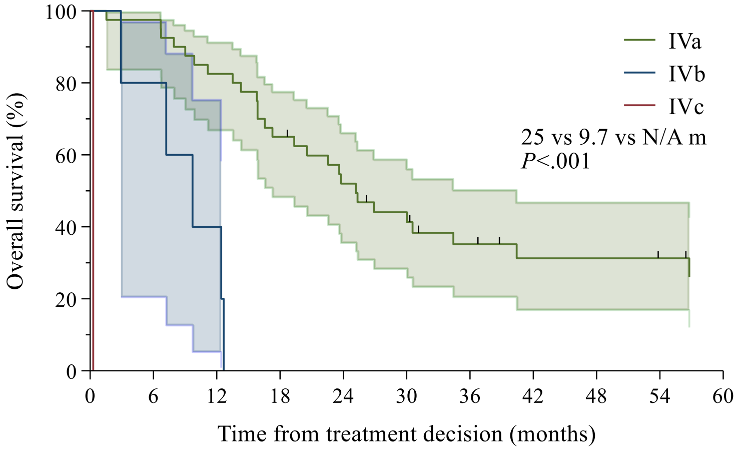

Number at risk											
IVa	40	39	33	26	20	16	11	8	8	7	5
IVb	5	4	2	0	0	0	0	0	0	0	0
IVc	1	0	0	0	0	0	0	0	0	0	0

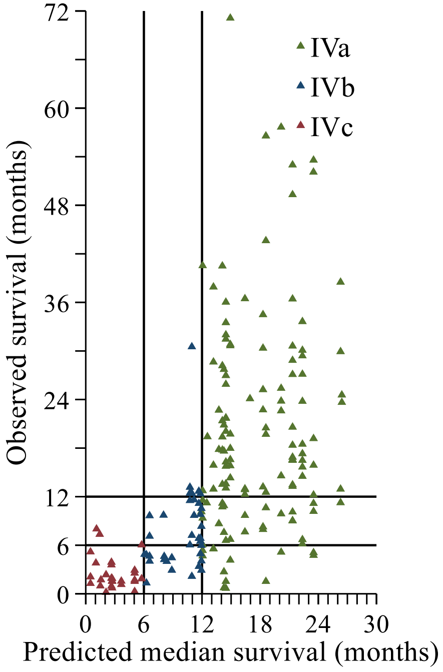

Fig. S5. Scatterplot of the predicted median overall survival time against observed overall survival by the Helsinki University Hospital Working Formulation stages IVa (predicted median survival >12 months), IVb (predicted <12-6 months), and IVc (predicted <6 months.

A

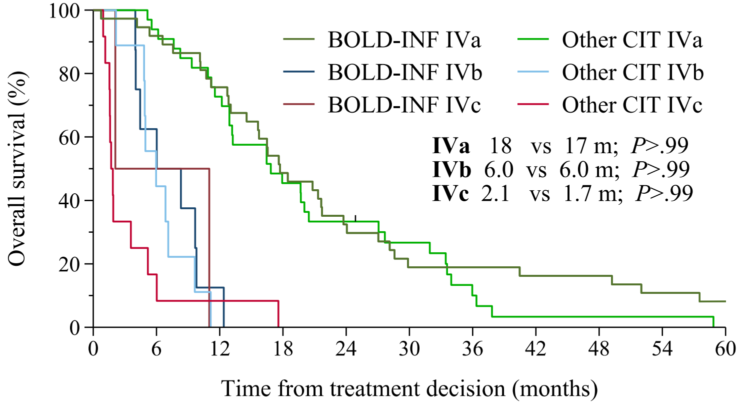

Number at risk											
BOLD-INF IVa	37	34	28	18	12	7	7	6	6	4	3
BOLD-INF IVb	8	5	1	0	0	0	0	0	0	0	0
BOLD-INF IVc	2	1	0	0	0	0	0	0	0	0	0
Other CIT IVa	2	1	1	0	0	0	0	0	0	0	0
Other CIT IVb	33	31	24	15	11	8	3	1	1	1	0
Other CIT IVc	9	4	0	0	0	0	0	0	0	0	0

Fig. S6. Kaplan-Meier graph of overall survival (OS) from decision to treat to death for (A) chemoimmunotherapy with interferon or interleukin (CIT) according to the chemotherapeutic combinations: BOLD (bleomycin + vincristine + lomustine +dacarbazine) in combination with interferon and other CITs according to the Helsinki University Hospital Working Formulation (WF); (B) interferon or interleukin (IFN/IL) against best supportive care (BSC) according to the WF; (C) check-point inhibitor (CPI) against BSC according to the WF. Median OS is given, and *P* value calculated by log-rank test and adjusted with Bonferroni correction.

B

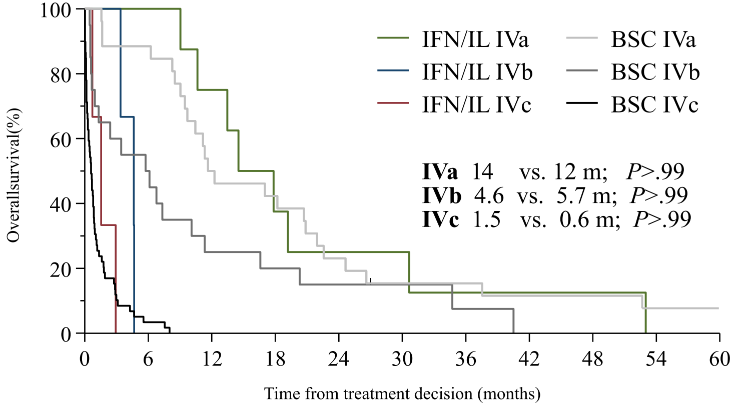

Number at risk

IFN/IL IVa	8	8	6	3	2	2	1	1	1	0	0
IFN/IL IVb	3	0	0	0	0	0	0	0	0	0	0
IFN/IL IVc	3	0	0	0	0	0	0	0	0	0	0
BSC IVa	26	23	13	11	6	4	4	3	3	2	2
BSC IVb	20	10	5	4	3	2	1	0	0	0	0
BSC IVc	59	2	0	0	0	0	0	0	0	0	0

C

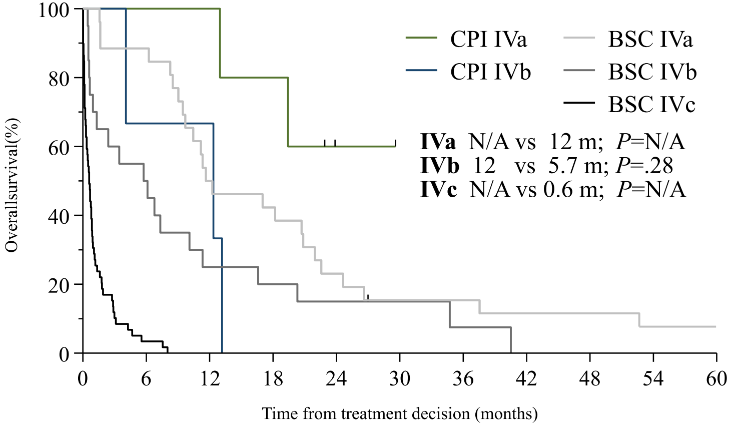

Number at risk

CPI IVa	5	5	5	4	1	0	0	0	0	0	0
CPI IVb	3	2	2	0	0	0	0	0	0	0	0
BSC IVa	26	23	13	11	6	4	4	3	3	2	2
BSC IVb	20	10	5	4	3	2	1	0	0	0	0
BSC IVc	59	2	0	0	0	0	0	0	0	0	0

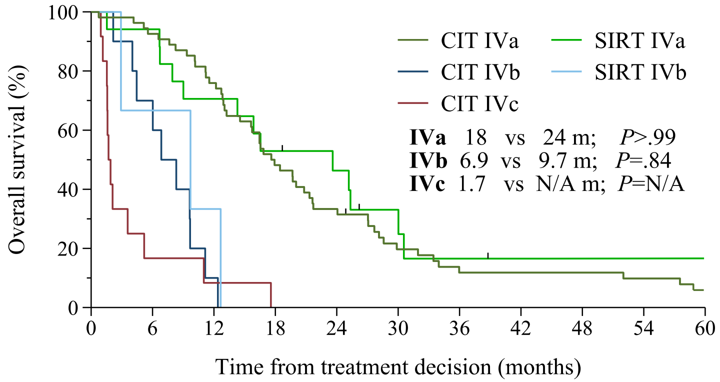

Number at risk

CIT IVa	54	50	41	26	18	10	6	6	6	5	3
CIT IVb	10	7	1	0	0	0	0	0	0	0	0
CIT IVa	12	2	1	0	0	0	0	0	0	0	0
SIRT IVa	17	16	12	9	7	4	2	1	1	1	1
SIRT IVb	3	2	1	0	0	0	0	0	0	0	0

Fig. S7. Kaplan-Meier graph of overall survival (OS) from treatment decision to death for chemoimmunotherapy with interferon or interleukin (CIT) against selective internal radiation therapy (SIRT) staged with Helsinki University Hospital Working Formulation for patients with only hepatic metastases. Median OS is given, and *P* value is calculated by log-rank test and adjusted with Bonferroni correction.

A

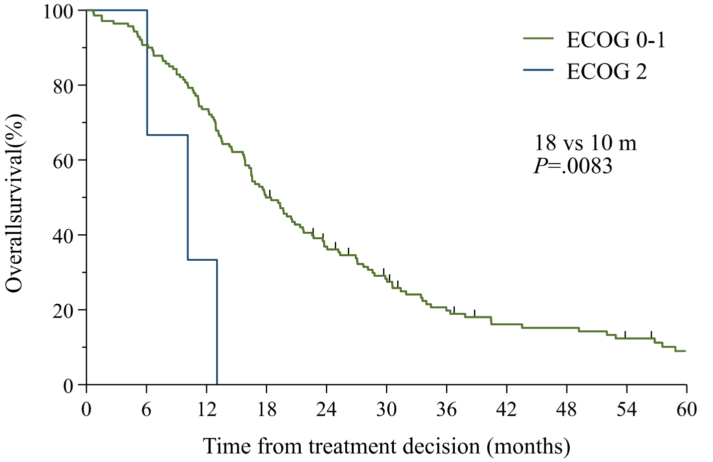

Number at risk											
ECOG 0-1 IVa	140	127	103	70	49	35	23	17	16	12	8
ECOG 2 IVa	3	3	1	0	0	0	0	0	0	0	0

Fig. S8. Kaplan-Meier graph of overall survival (OS) from treatment decision to death according to components of Helsinki University Hospital Working Formulation and staged by it (A) Eastern Cooperative Oncology Group (ECOG) performance status by stage IVa; (B) by IVb; (C) by IVc; (D) plasma or serum level of alkaline phosphatase by stage IVa; (E) by IVb; (F) by IVc; (G) the largest diameter of the largest metastasis by TNM M1 classification by stage IVa; (H) by IVb; and (I) by IVc. Median OS is given, and *P* value is calculated by log-rank test for unordered and log-rank test for trend for ordered categories.
UNL, upper normal limit.

B

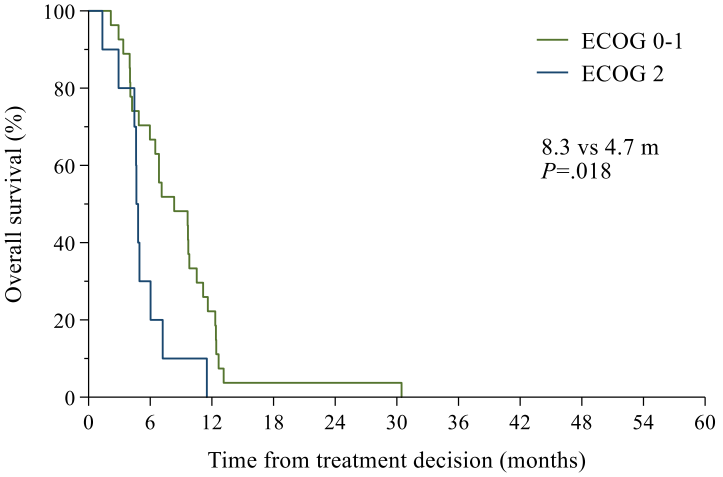

Number at risk											
ECOG 0-1 IVb	27	18	6	1	1	1	0	0	0	0	0
ECOG 2 IVb	10	3	0	0	0	0	0	0	0	0	0

C

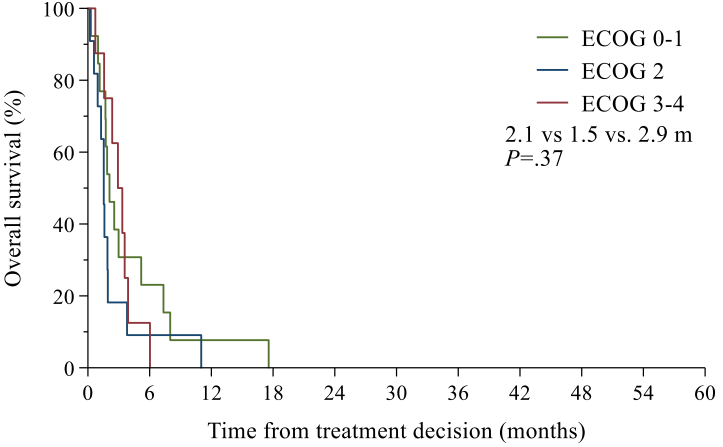

Number at risk											
ECOG 0-1 IVc	13	3	1	0	0	0	0	0	0	0	0
ECOG 2 IVc	11	1	0	0	0	0	0	0	0	0	0
ECOG 3-4 IVc	8	1	0	0	0	0	0	0	0	0	0

D

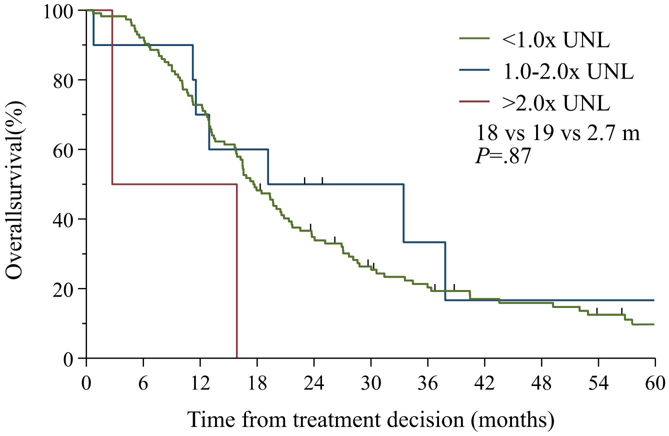

Number at risk											
<1.0x UNL IVa	114	105	83	55	38	27	20	15	14	10	7
1.0-2.0x UNL IVa	10	9	7	6	4	3	2	1	1	1	1
>2.0x UNL IVa	2	1	1	0	0	0	0	0	0	0	0

E

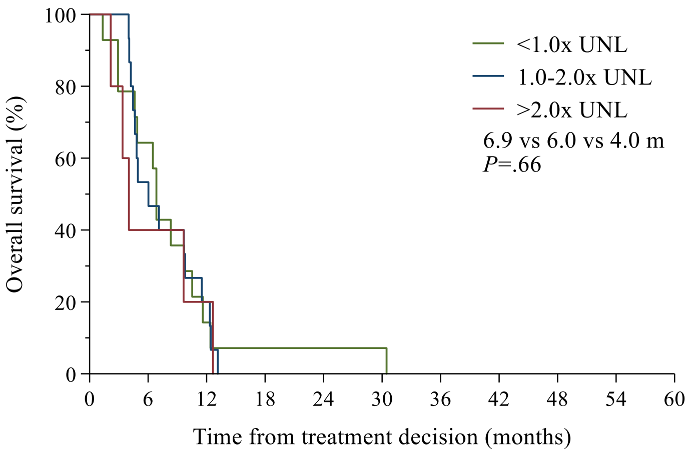

Number at risk											
<1.0x UNL IVb	14	9	2	1	1	1	0	0	0	0	0
1.0-2.0x UNL IVb	15	8	3	0	0	0	0	0	0	0	0
>2.0x UNL IVb	5	2	1	0	0	0	0	0	0	0	0

F

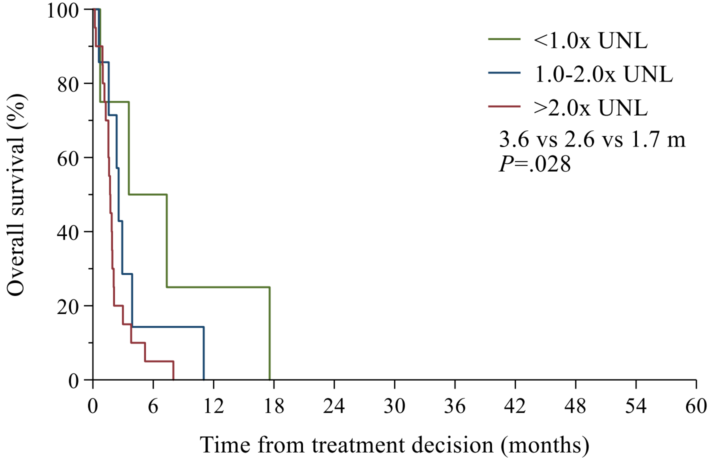

Number at risk											
<1.0x UNL IVc	4	2	1	0	0	0	0	0	0	0	0
1.0-2.0x UNL IVc	7	1	0	0	0	0	0	0	0	0	0
>2.0x UNL IVc	20	1	0	0	0	0	0	0	0	0	0

G

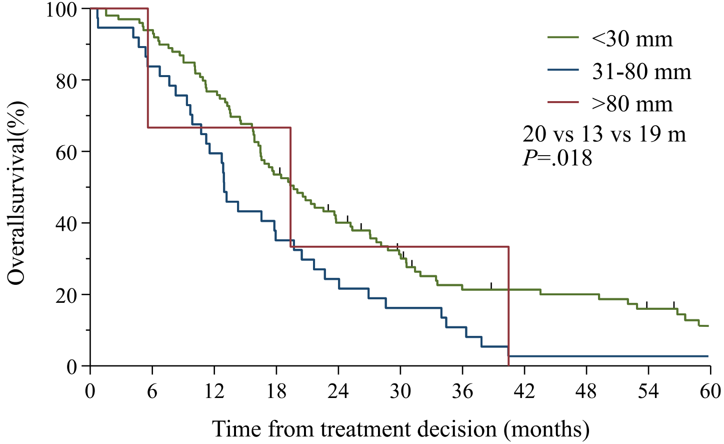

Number at risk											
<30 mm IVa	99	93	76	53	38	27	17	16	15	11	7
31-80 mm IVa	37	31	22	13	9	6	4	1	1	1	1
>80 mm IVa	3	2	2	2	1	1	1	0	0	0	0

H

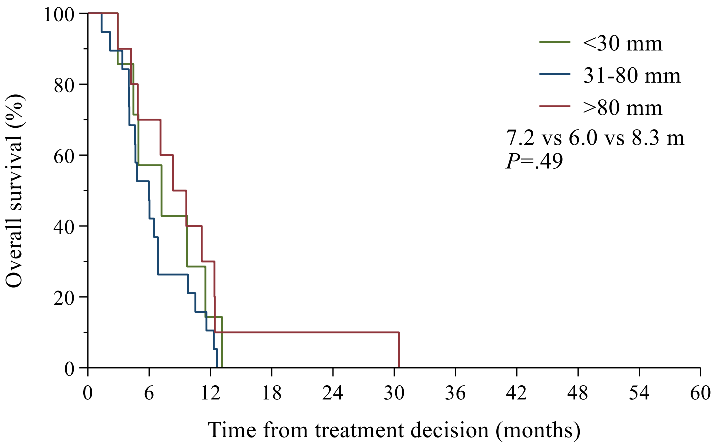

Number at risk											
<30 mm IVb	7	4	1	0	0	0	0	0	0	0	0
31-80 mm IVb	19	9	2	0	0	0	0	0	0	0	0
>80 mm IVb	10	7	3	1	1	1	0	0	0	0	0

I

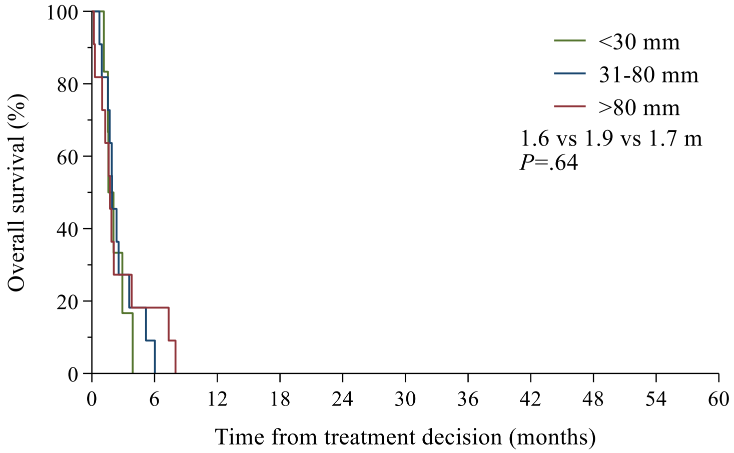

Number at risk											
<30 mm IVc	6	0	0	0	0	0	0	0	0	0	0
31-80 mm IVc	11	1	0	0	0	0	0	0	0	0	0
>80 mm IVc	11	2	0	0	0	0	0	0	0	0	0

A

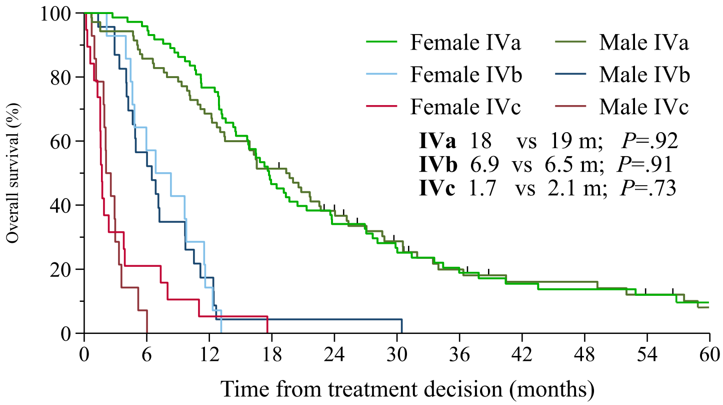

Number at risk

Female IVa	73	70	56	34	24	18	12	9	8	6	4
Female IVb	14	8	2	0	0	0	0	0	0	0	0
Female IVc	19	4	1	0	0	0	0	0	0	0	0
Male IVa	70	60	48	36	25	17	11	8	8	6	4
Male IVb	23	13	4	1	1	1	0	0	0	0	0
Male IVc	14	1	0	0	0	0	0	0	0	0	0

Fig. S9. Kaplan-Meier graph of overall survival (OS) from treatment decision to death staged by Helsinki University Hospital Working Formulation according to (A) gender; (B) age tertiles IVa; (C) IVb; (D) IVc; (E) symptoms; (F) distant metastasis-free interval <2.0, 2.0-3.5, 3.5-5.0, and >5.0 years stage IVa; (G) IVb; (H) IVc; (I) sites of metastases IVa; (J) IVb; (K) IVc; (L) plasma or serum level lactate dehydrogenase by stage IVa ; (M) IVb; and (N) IVc. Median OS is given, and *P* value is calculated by log-rank test for unordered and log-rank test for trend for ordered categories.
UNL, upper normal limit.

B

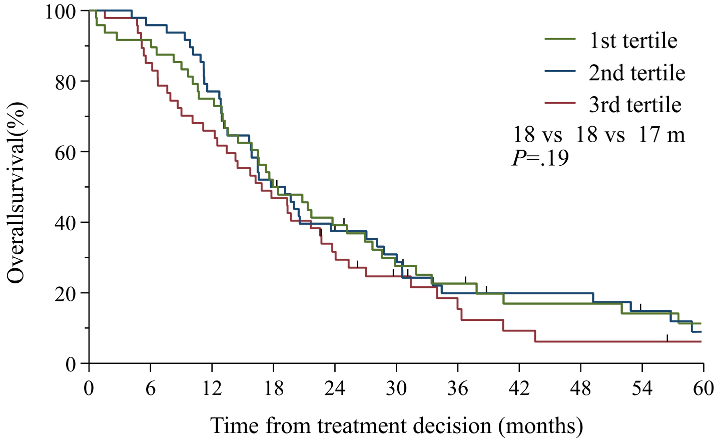

Number at risk											
1st tertile IVa	48	44	36	24	18	12	9	6	6	5	4
2nd tertile IVa	48	46	37	24	17	14	9	8	8	5	3
3rd tertile IVa	47	40	31	22	14	9	5	3	2	2	1

C

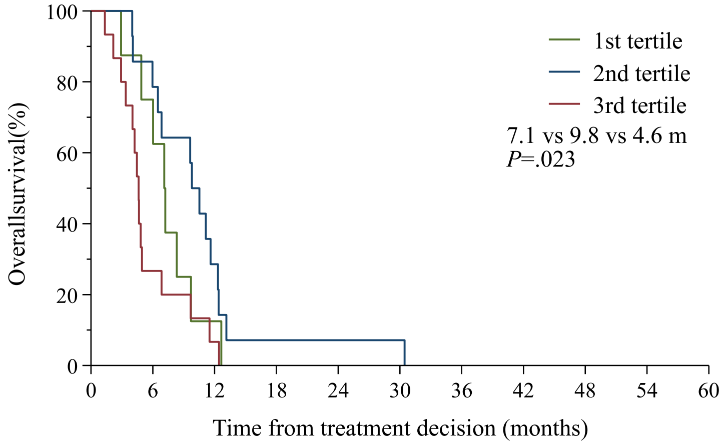

Number at risk											
1st tertile IVb	8	6	1	0	0	0	0	0	0	0	0
2nd tertile IVb	14	11	4	1	1	1	0	0	0	0	0
3rd tertile IVb	15	4	1	0	0	0	0	0	0	0	0

D

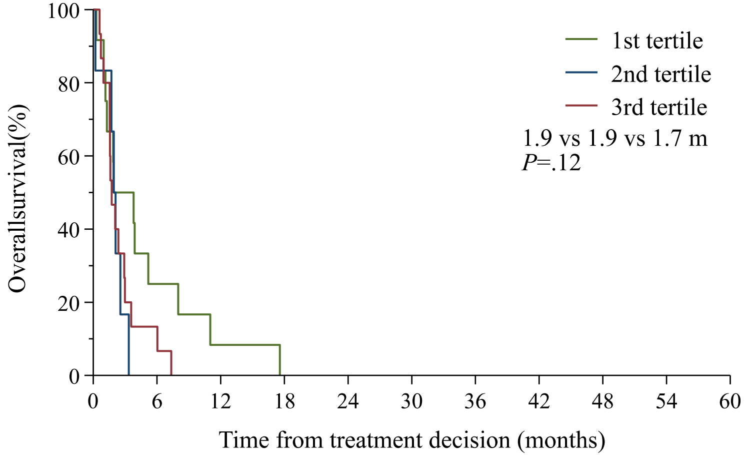

Number at risk

1st tertile IVc	12	3	1	0	0	0	0	0	0	0	0
2nd tertile IVc	6	0	0	0	0	0	0	0	0	0	0
3rd tertile IVc	15	2	0	0	0	0	0	0	0	0	0

E

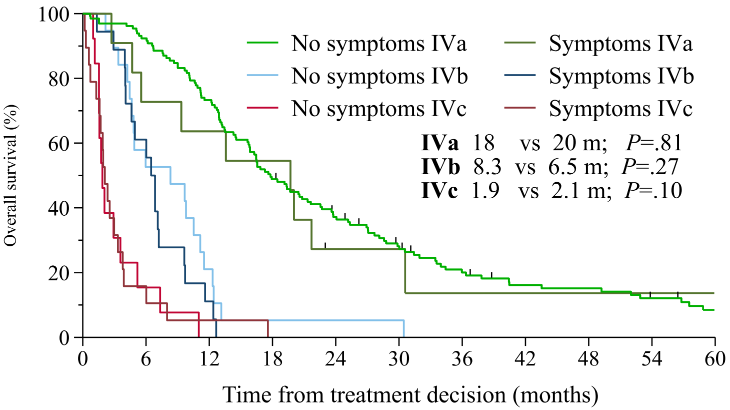

Number at risk

No symptoms IVa	131	121	96	64	47	33	22	16	15	11	7
No symptoms IVb	19	10	4	1	1	1	0	0	0	0	0
No symptoms IVc	13	2	0	0	0	0	0	0	0	0	0
Symptoms IVa	2	0	0	0	0	0	0	0	0	0	0
Symptoms IVb	11	8	7	6	2	2	1	1	1	1	1
Symptoms IVc	18	11	2	0	0	0	0	0	0	0	0

F

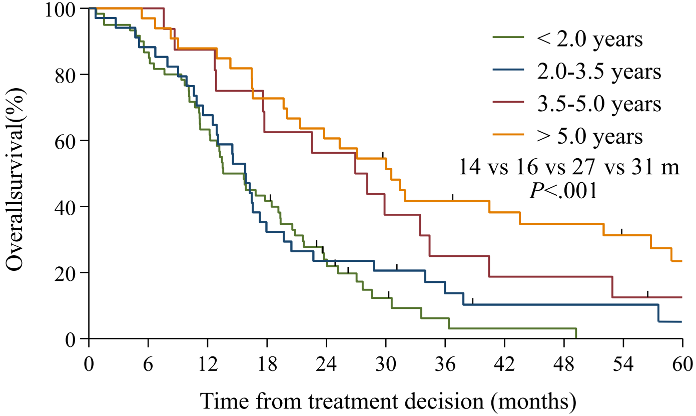

Number at risk											
< 2.0 years IVa	60	52	38	25	12	5	2	1	1	0	0
2.0-3.5 years IVa	34	30	23	11	8	7	4	2	2	2	1
3.5-5.0 years IVa	16	16	14	10	9	6	4	3	3	2	1
> 5.0 years IVa	33	32	29	24	20	17	13	11	10	8	6

G

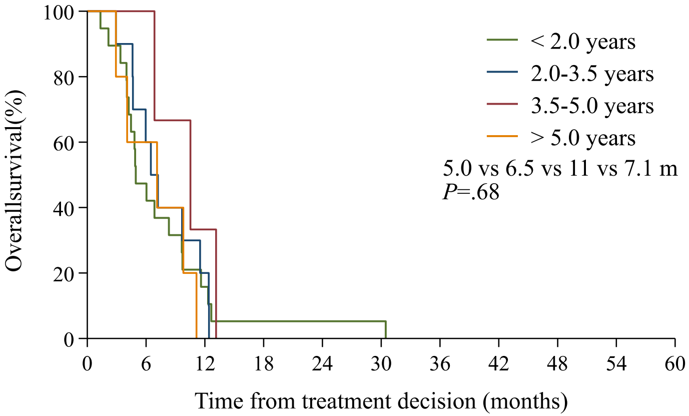

Number at risk

< 2.0 years IVb	19	9	3	1	1	1	0	0	0	0	0
2.0-3.5 years IVb	10	6	2	0	0	0	0	0	0	0	0
3.5-5.0 years IVb	3	3	1	0	0	0	0	0	0	0	0
> 5.0 years IVb	5	3	0	0	0	0	0	0	0	0	0

H

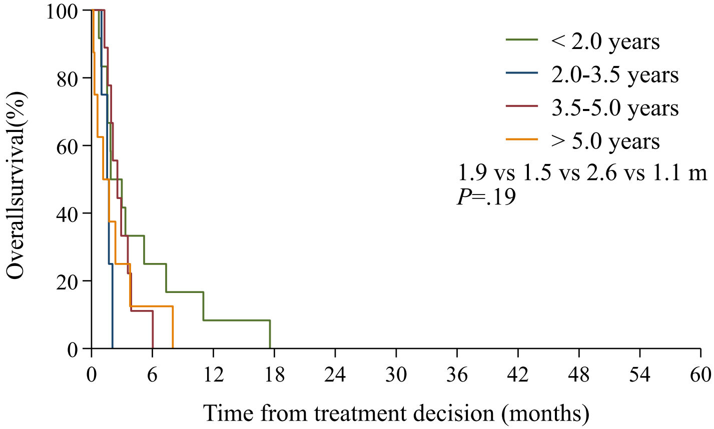

Number at risk											
< 2.0 years IVc	12	3	1	0	0	0	0	0	0	0	0
2.0-3.5 years IVc	4	0	0	0	0	0	0	0	0	0	0
3.5-5.0 years IVc	9	1	0	0	0	0	0	0	0	0	0
> 5.0 years IVc	8	1	0	0	0	0	0	0	0	0	0

I

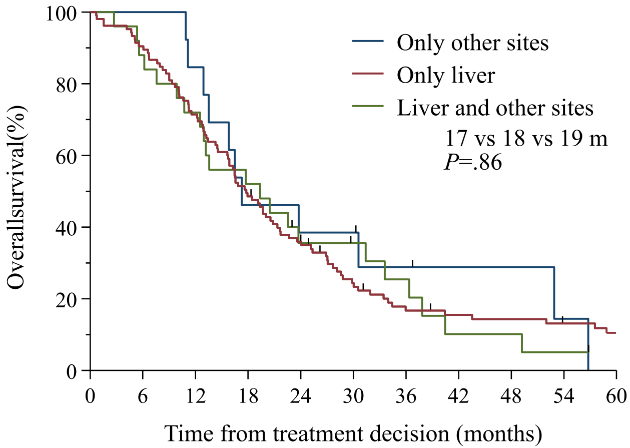

Number at risk											
Only other sites IVa	13	13	11	6	5	5	3	2	2	1	0
Only liver IVa	105	95	75	51	36	23	15	13	12	10	8
Liver and other sites IVa	25	22	18	13	8	7	5	2	2	1	0

J

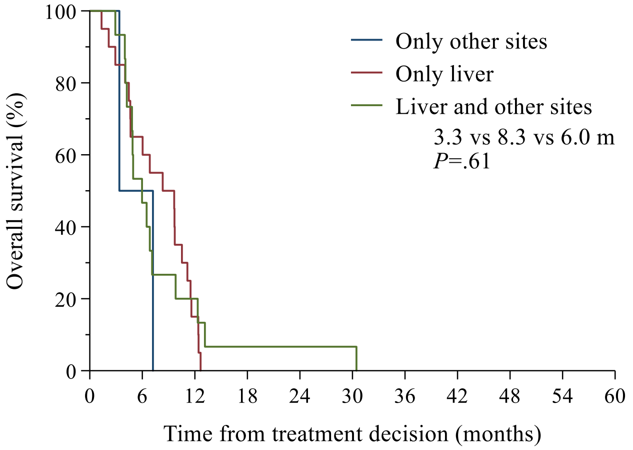

Number at risk											
Only other sites IVb	2	1	0	0	0	0	0	0	0	0	0
Only liver IVb	20	13	3	0	0	0	0	0	0	0	0
Liver and other sites IVb	15	7	3	1	1	1	0	0	0	0	0

K

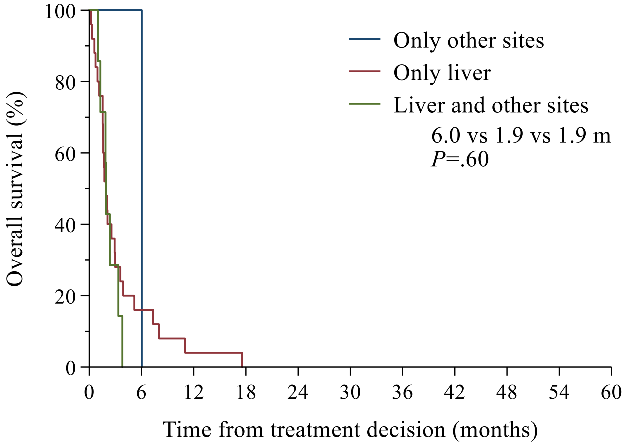

Number at risk											
Only other sites IVc	1	1	0	0	0	0	0	0	0	0	0
Only liver IVc	25	4	1	0	0	0	0	0	0	0	0
Liver and other sites IVc	7	0	0	0	0	0	0	0	0	0	0

L

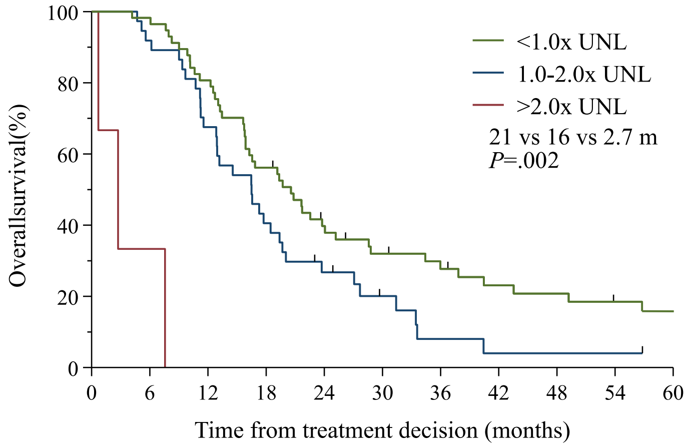

Number at risk											
<1.0x UNL IVa	57	56	46	32	21	16	13	10	9	7	6
1.0-2.0x UNL IVa	37	34	25	15	9	5	2	1	1	1	0
>2.0x UNL IVa	3	1	0	0	0	0	0	0	0	0	0

M

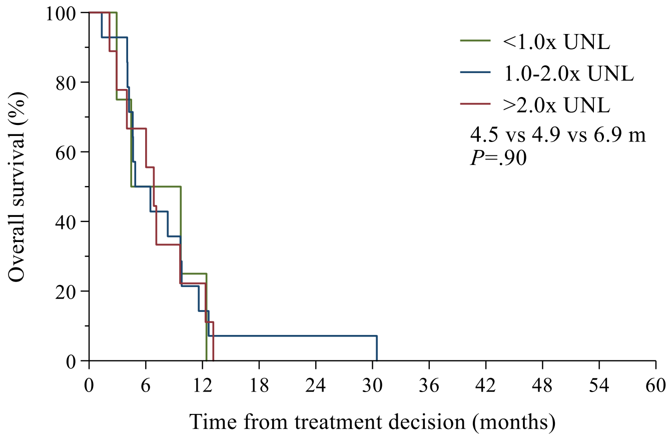

Number at risk											
<1.0x UNL IVb	4	2	1	0	0	0	0	0	0	0	0
1.0-2.0x UNL IVb	14	7	2	1	1	1	0	0	0	0	0
>2.0x UNL IVb	9	6	2	0	0	0	0	0	0	0	0

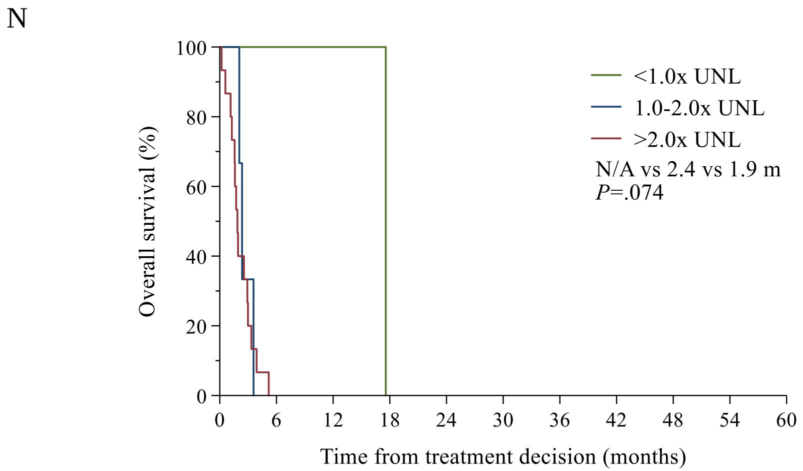

Number at risk											
<1.0x UNL IVc	1	1	1	0	0	0	0	0	0	0	0
1.0-2.0x UNL IVc	3	0	0	0	0	0	0	0	0	0	0
>2.0x UNL IVc	15	0	0	0	0	0	0	0	0	0	0
