# Supplementary material for: Impact of staging on survival outcomes: a nationwide real-world cohort study of metastatic uveal melanoma
Source: Melanoma Res. 2021 Mar 5;31(3):224–31. doi: 10.1097/CMR.0000000000000728 (PMC8081447; doi:10.1097/CMR.0000000000000728)
Supplement: Supplementary file 2 [file mr-31-224-s002.pdf]

Supplemental Digital Content 2. Supplementary Text and Tables S1-S7.
Supplementary Texts for Methods
Supplementary Text S1.

Verification of Metastases
We adapted definitions of the Collaborative Ocular Melanoma Study (COMS) to ascertain whether metastatic UM was present [1,2]. If the original pathology report mentioned moderate to heavy melanin or either HMB-45 or MelanA/MART-1 immunopositivity, the code was “dead with melanoma metastases, confirmed metastases”. If it mentioned none of these characteristics and was not a fine-needle aspiration biopsy (FNAB), we (TTK, ESR) obtained the original specimen for review. If melanin was equivocal, we performed HMB-45, MelanA/MART-1, and pan-cytokeratin immunostainings [2]. If only a FNAB was available or no histopathologic confirmation had been sought, but clinical findings (hepatomegaly, elevated liver function tests, liver imaging) were consistent with progressive metastases, the code was “suspected metastases”. If the death certificate specified metastatic melanoma as cause of death (CoD) but clinical data were inconclusive, or the specified CoD was other than metastases, but clinical findings were consistent with metastases, it was “possible metastases”. If histopathology was not diagnostic of metastatic melanoma and clinical data were inconclusive, the code was “dead, not consistent with melanoma metastases” and the patient was excluded.

Review of non-malignant cause of death according to the original death certificate
No patients died of non-malignant causes according to their death certificates signed by general practitioners.

Review of second malignancies according to the original death certificate
A second malignancy was recorded as the CoD for one patient by a general practitioner. The patient had cutaneous melanoma as CoD, but a core needle biopsy (CNB) from the liver had abundant melanin pigment and in the laparoscopy the liver was covered with small dark metastases, no skin lesions or cutaneous cancer whatsoever were ever mentioned in his charts and the metastases progressed. The patient was coded with uveal melanoma metastases, confirmed.

Central review of histopathologic findings not consistent with melanoma metastases according to the original pathology report
Three patients had histopathologic findings not consistent with melanoma metastases according to the original pathology report. The first patient was diagnosed with hypoechoic suspected metastasis and normal chest X-ray one year after the diagnosis of the primary uveal melanoma. A CNB from the liver showed fibrosis, but six months later the suspected metastasis had progressed and a FNAB showed no atypia, but erythrocytes and steatosis. Six months later also chest X-ray showed suspected metastases. The second patient was diagnosed with two hypoechoic suspected metastases in the liver four years after the diagnosis of the primary uveal melanoma. A subsequent CNB from the liver was negative with unspecific inflammation confirmed by the central review. A computed tomography (CT) was done 2.5 months after the US and it detected four hypervascular metastases that progressed. The third patient was diagnosed with a local prostate carcinoma 3 years after the primary uveal melanoma, and he was treated with neoadjuvant and radical radiotherapy. Four months later a liver metastasis, diagnosed by upper abdominal ultrasonography and CT with no bone metastases, was treated with stereotactic radiofrequency ablation. A CNB from the liver showed only steatosis and fibrosis. The remission has lasted for two years. All patients were coded with melanoma metastases, possible.

After review, 71% had confirmed metastases, 7% suspected metastases, and 2% possible metastases.

Table S1.
Categorization of stage IV metastatic uveal melanoma into stage IVa, IVb, and IVc according to the predicted median survival, in months, based on Eastern Cooperative Oncology Group (ECOG) performance status, also called WHO performance status, serum or plasma alkaline phosphatase level, and the largest dimension of the largest metastasis. Stage IVa corresponds to predicted overall survival of ≥12 months, IVb <12-6 months, and IVc <6 months, adapted from [3]. The performance index was divided into 3 categories: ECOG/WHO performance status 0; ECOG/WHO performance status 1-2; and performance status ECOG/WHO 3-4. The model assigns patients in prognostic groups based on predicted overall survival, calculated on the basis of the covariates of each patient and the baseline survivor function of the original Cox multivariable regression: stage IVa corresponds to predicted overall survival of ≥12 months, IVb <12–6 months, and IVc <6 months. The schematic table is composed identically and is adapted from [3]. An on-line calculator to assign the stage is available at: http://www.prognomics.org/huhwf.aspx

		Largest Dimension of Largest Metastasis (mm)				
ECOG	AP Level x UNL	20	40	60	80	100
0	1.0	**A** 14	12	11	8	7
	1.4	13	12	8	8	6
	1.8	12	11	8	6	5
	2.2	**B** 11	8	7	6	4
1-2	1.0	11	8	6	6	4
	1.4	8	8	6	4	3
	1.8	8	6	**C** 5	4	2
	2.2	7	6	4	3	2

AP, alkaline phosphatase; ECOG, Eastern Cooperative Oncology Group performance status; UNL, upper normal limit.

Table S2.
Baseline characteristics of the entire cohort managed with active treatment, and stratification by the Helsinki University Hospital Working Formulation stage.[a]

Variable	All patients N=216	Stage IVa N =143 (67%)	Stage IVb N =37 (17%)	Stage IVc N =33 (15%)
Gender, N (%)[b]				
Female	106 (49)	73 (51)	14 (38)	19 (58)
Male	110 (51)	70 (49)	23 (62)	14 (42)
TNM stage, N (%)				
I	18 (8)	11 (8)	3 (8)	4 (12)
IIA	37 (17)	24 (17)	6 (16)	7 (21)
IIB	44 (20)	30 (21)	7 (19)	7 (21)
IIIA	56 (26)	39 (27)	12 (32)	3 (9)
IIIB	36 (17)	22 (15)	5 (14)	8 (24)
IIIC	2 (1)	0 (0)	1 (3)	1 (3)
IV	23 (11)	17 (12)	3 (8)	3 (9)
Primary tumour extent, N (%)				
Limited to choroid	120 (56)	82 (57)	18 (49)	20 (61)
With ciliary body involvement	90 (42)	58 (41)	17 (46)	12 (36)
Extraocular extension	6 (3)	3 (2)	2 (5)	1 (3)
Follow-up for metastases, N (%)				
None	1 (0)	0 (0)	0 (0)	1(3)
Irregular	2 (1)	0 (0)	0 (0)	2 (6)
Regular	213 (99)	143 (100)	37 (100)	30 (91)
Symptoms from metastasis, N (%)				
No	164 (76)	131 (92)	19 (51)	13 (39)
Yes	50 (23)	11 (8)	18 (49)	19 (58)
Unknown	2 (1)	1 (1)	0 (0)	1 (3)
Distant metastasis-free interval, N (%)				
<2.0 years	94 (44)	60 (42)	19 (51)	12 (36)
2.0-3.5 years	48 (22)	34 (24)	10 (27)	4 (12)
>3.5 years	74 (34)	49 (34)	8 (22)	17 (52)
Histologic confirmation, N (%)				
Yes	174 (81)	119 (83)	28 (76)	24 (73)
No	42 (19)	24 (17)	9 (24)	9 (27)
Location of metastases at the time of diagnosis, N (%)				
Liver only	152 (70)	105 (73)	20 (54)	25 (76)
Liver and other sites	47 (22)	25 (18)	15 (41)	7 (21)
Other sites only	17 (8)	13 (9)	2 (5)	1 (3)
Median largest diameter of the largest metastasis, mm (range, IQR)	30 (2-196, 18-50)	23 (2-90, 16-34)	57 (10-125, 41-80)	65 (13-196, 35-118)
TNM M1 category, N (%)				
≤30 mm (M1a)	113 (52)	99 (69)	7 (19)	6 (18)
31-80 mm (M1b)	67 (31)	37 (26)	19 (51)	11 (33)
>80 mm (M1c)	24 (11)	3 (2)	10 (27)	11 (33)
Unknown	12 (6)	4 (3)	1 (3)	5 (15)

Liver function tests, N (%)				
AP				
<1.0 x UNL	133 (62)	114 (80)	14 (38)	4 (12)
1.0-2.0 x UNL	33 (15)	10 (7)	15 (41)	7 (21)
>2.0 x UNL	28 (13)	2 (1)	5 (14)	20 (61)
Unknown	22 (10)	17 (12)	3 (8)	2 (6)
LDH				
<1.0 x UNL	61 (28)	57 (40)	4 (11)	1 (3)
1.0-2.0 x UNL	56 (26)	37 (26)	14 (38)	3 (9)
>2.0 x UNL	28 (13)	3 (2)	9 (24)	15 (45)
Unknown	71 (33)	46 (32)	10 (27)	14 (42)
AST				
<1.0 x UNL	97 (45)	73 (51)	20 (54)	5 (15)
1.0-2.0 x UNL	26 (12)	16 (11)	6 (16)	3 (9)
>2.0 x UNL	22 (10)	4 (3)	4 (11)	13 (39)
Unknown	71 (33)	50 (35)	7 (19)	12 (36)
ALT				
<1.0 x UNL	137 (63)	101 (71)	24 (65)	11 (33)
1.0-2.0 x UNL	36 (17)	18 (13)	6 (16)	12 (36)
>2.0 x UNL	17 (8)	5 (3)	4 (11)	7 (21)
Unknown	26 (12)	19 (13)	3 (8)	3 (9)
Performance status, N (%)[c]				
0-1	182 (84)	140 (98)	27 (73)	13 (39)
2	25 (12)	3 (2)	10 (27)	11 (33)
3-4	8 (4)	0 (0)	0 (0)	8 (24)
Unknown	1 (0)	0 (0)	0 (0)	1 (3)
Age at treatment decision, median (range, IQR), y	64 (21-86, 57-71)	63 (21-86, 56-71)	68 (34-82, 60-72)	65 (24-85, 55-73)

Abbreviations: ALT, alkaline aminotransferase; AP, alkaline phosphatase; AST, aspartate aminotransferase; IQR, interquartile range; LDH, lactate dehydrogenase; UNL, upper normal limit.

[a] Stage IVa corresponds to predicted overall survival of ≥12 months, IVb <12-6 months, and IVc <6 months. Three patients could not be staged.

[b] Binomial test, *P*=0.51.

[c] Eastern Cooperative Oncology Group performance status.

Table S3.
Reasons for a delay of more than 90 days from diagnosis of metastasis to treatment decision.

Reason	N
Initially negative FNAB or CNB result	21
Unfavorable performance status	0
Patient preference	2
Administrative reasons	1 [a]
Considered for SIRT but eventually not eligible	1
Not specified	34

Abbreviations: CNB, core-needle biopsy; FNAB, fine-needle aspiration biopsy; SIRT, selective internal radiation therapy.
[a] Waiting for a trial opening.

Table S4.
Agreement between observed survival from treatment decision and the prediction according to the Helsinki University Hospital Working Formulation stage.[a]

Working Formulation Stage	Observed overall survival		
	≥12 months<br>N (%)	<12-6 months<br>N (%)	<6 months<br>N (%)
IVa	104 (73)	26 (18)	13 (9)
IVb	6 (16)	15 (41)	16 (43)
IVc	1 (3)	4 (12)	28 (85)

[a] Stage IVa corresponds to predicted overall survival of ≥12 months, IVb <12-6 months, and IVc <6 months; three patients could not be staged.
Weighted kappa, 0.549.

Table S5.

Type of surgical intervention for 19 patients whose 1st line treatment was surgery. Target organs are listed in the footnotes.

Surgical intervention	Number of patients (%)
Resection	19[a]
Radical resection (R0)	8[b]

[a] Liver 10, lung 2, breast, parotis, brain, subcutaneous tissue, spleen, muscle, and kidney one each.

[b] Liver 6, lung and kidney one each.

Table S6.
Summary table of patients with only hepatic metastases who received chemoimmunotherapy with interferon or interleukin (CIT), conventional chemotherapy (CHT), selective internal radiation therapy (SIRT), or local treatment as first-line treatment.

Variable	CIT N=78	CHT N=27	SIRT N=20	Local treatment N=34
Gender, N (%)				
Female	38 (48)	12 (44)	6 (30)	14 (41)
Male	40 (51)	15 (56)	14 (70)	20 (59)
Age, median (range, IQR)				
Primary tumour	58 (28-81, 51-67)	67 (19-85, 59-74)	58 (27-82, 54-63)	57 (27-82, 53-63)
Treatment decision	61 (32-83, 56-70)	71 (24-86, 63-77)	63 (33-82, 56-70)	60 (33-83, 56-70)
Death	64 (34-84, 58-72)	71 (24-87, 63-87)	64 (34-84, 57-71)	64 (34-86, 57-72)
Primary tumour extent, N (%)				
Limited to choroid	43 (55)	18 (67)	11 (55)	20 (59)
With ciliary body involvement	32 (41)	8 (30)	9 (45)	14 (41)
Extraocular extension	3 (4)	1 (4)	0 (0)	0 (0)
TNM stage, N (%)				
I	5 (6)	1 (4)	3 (15)	4 (12)
IIA	11 (14)	7 (26)	4 (20)	8 (24)
IIB	18 (23)	8 (30)	3 (15)	6 (18)
IIIA	20 (26)	2 (7)	5 (25)	10 (29)
IIIB	9 (12)	7 (26)	3 (15)	4 (12)
IIIC	1 (1)	0 (0)	0 (0)	0 (0)
IV	14 (18)	2 (7)	2 (10)	2 (6)
Distant metastasis-free interval, N (%)				
<2.0 years	41 (53)	10 (37)	8 (40)	10 (29)
2.0-3.5 years	13 (17)	8 (30)	5 (25)	8 (24)
>3.5 years	24 (31)	9 (33)	7 (35)	16 (47)
Follow-up for metastases, N (%)				
None	0 (0)	0 (0)	0 (0)	0 (0)
Irregular	0 (0)	1 (4)	0 (0)	0 (0)
Regular	78 (100)	26 (96)	20 (100)	34 (100)
Symptoms from metastasis, N (%)				
No	61 (78)	18 (67)	18 (90)	30 (89)
Yes	16 (21)	9 (33)	2 (10)	4 (12)
Unknown	1 (1)	0 (0)	0 (0)	0 (0)
Median largest diameter of the largest metastasis, mm (range, IQR)	30 (9-160, 20-50)	26 (9-182, 15-56)	23 (2-125, 11-30)	27 (2-130, 15-34)
TNM M1 category, N (%)				
≤30 mm (M1a)	39 (50)	14 (52)	16 (80)	24 (71)
31-80 mm (M1b)	28 (36)	7 (26)	3 (15)	7 (21)
>80 mm (M1c)	6 (8)	4 (15)	1 (5)	3 (9)
Unknown	5 (6)	2 (7)	0 (0)	0 (0)
Alkaline phosphatase, N (%)				
<1.0 x UNL	49 (63)	17 (63)	13 (93)	22 (65)
1.0-2.0 x UNL	11 (14)	4 (15)	0 (0)	2 (5)
>2.0 x UNL	12 (15)	5 (19)	1 (7)	3 (8)
Unknown	6 (8)	1 (4)	0 (0)	7 (21)

Performance status, N (%)[a]				
0-1	67 (86)	20 (74)	20 (100)	34 (100)
2	9 (12)	5 (19)	0 (0)	0 (0)
3-4	2 (3)	1 (4)	0 (0)	0 (0)
Unknown	0 (0)	1 (4)	0 (0)	0 (0)
WF stage[b]				
IVa	54 (69)	14 (52)	17 (85)	29 (85)
IVb	10 (13)	4 (15)	3 (15)	4 (12)
IVc	12 (15)	9 (33)	0 (0)	1 (3)
Unknown	2 (3)	0 (0)	0 (0)	0 (0)

Abbreviations: ALT, alkaline aminotransferase; AST, aspartate aminotransferase; CHT, conventional chemotherapy; CIT, chemoimmunotherapy with interferon or interleukin; IQR, interquartile range; LDH, lactate dehydrogenase; SIRT, selective internal radiation therapy; UNL, upper normal limit; WF, Helsinki University Hospital Working Formulation; three patients could not be staged in the entire cohort.

[a] Eastern Cooperative Oncology Group performance status.

[b] Stage IVa corresponds to predicted overall survival of ≥12 months, IVb <12-6 months, and IVc <6 months.

Table S7.
Cox proportional hazards regression of overall survival after treatment decision.

Variable	Regression coefficient (SE)	Wald χ^2	P	Hazard ratio (95% CI)
Univariable analysis				
WF stage				
IVa	Reference			1.0
IVb	1.18 (0.22)	30.25	<0.001	3.26 (2.14-4.98)
IVc	1.92 (0.24)	66.25	<0.001	6.84 (4.30-10.86)
Gender				
Female	Reference			1.0
Male	0.19 (0.14)	1.76	0.19	1.21 (0.91-1.60)
Presence of symptoms				
No	Reference			1.0
Yes	0.52 (0.17)	8.94	0.003	1.68 (1.20-2.36)
Age in tertiles, range				
1st tertile (21-59 years)	Reference			1.0
2nd tertile (59-68 years)	-0.26 (0.18)	2.07	0.15	0.77 (0.54-1.10)
3rd tertile (68-86 years)	-0.08 (0.17)	0.21	0.65	0.92 (0.66-1.30)
Distant metastasis-free interval				
<2.0 years	Reference			1.0
2.0-3.5 years	-0.17 (0.18)	0.90	0.34	0.84 (0.59-1.21)
>3.5 years	-0.23 (0.17)	1.74	0.19	0.80 (0.57-1.12)
Location of metastases at diagnosis				
Only other sites	Reference			1.0
Only liver	0.13 (0.27)	0.21	0.65	1.13 (0.66-1.94)
Liver and other sites	0.12 (0.30)	0.15	0.70	1.12 (0.62-2.03)
Lactate dehydrogenase				
<1.0 x UNL	Reference			1.0
1.0-2.0 x UNL	0.20 (0.20)	1.00	0.32	1.22 (0.82-1.82)
>2.0 x UNL	1.72 (0.29)	35.64	<0.001	5.55 (3.15-9.79)
Bivariable analysis				
Model 1: – 2 log likelihood = 733.51				
WF stage				
IVa	Reference			1.0
IVb	1.15 (0.22)	28.84	<0.001	3.18 (2.09-4.86)
IVc	1.94 (0.24)	65.75	<0.001	7.05 (4.41-11.3)
Gender				
Female	Reference			1.0
Male	0.19 (0.15)	1.66	0.20	1.21 (0.91-1.61)
Model 2: –2 log likelihood = 734.11				
WF stage				
IVa	Reference			1.0
IVb	1.21 (0.23)	28.94	<0.001	3.29 (2.11-5.11)
IVc	1.96 (0.25)	58.98	<0.001	6.77 (4.09-11.2)
Presence of symptoms				
No	Reference			1.0
Yes	-0.06 (0.19)	0.09	0.76	0.95 (0.65-1.39)
Model 3: –2 log likelihood = 733.30				
WF stage				
IVa	Reference			1.0
IVb	1.23 (0.22)	31.58	<0.001	3.42 (2.22-5.26)
IVc	1.94 (0.25)	61.78	<0.001	6.80 (4.19-11.0)
Age in tertiles				
1st tertile	Reference			1.0
2nd tertile	-0.09 (0.19)	0.21	0.65	0.89 (0.61-1.30)
3rd tertile	0.17 (0.18)	0.83	0.36	1.16 (0.81-1.66)

Model 4: –2 log likelihood = 733.93				
WF stage				
IVa	Reference			
IVb	1.17 (0.22)	29.27	<0.001	3.22 (2.11-4.92)
IVc	1.93 (0.24)	64.16	<0.001	6.90 (4.30-11.1)
Distant metastasis-free interval				
<2.0 years	Reference			
2.0-3.5 years	0.05 (0.19)	0.06	0.81	1.05 (0.72-1.52)
>3.5 years	-0.12 (0.17)	0.49	0.48	0.88 (0.63-1.25)
Model 5: –2 log likelihood = 734.22				
WF stage				
IVa	Reference			1.0
IVb	1.19 (0.22)	29.27	<0.001	3.30 (2.14-5.10)
IVc	1.91 (0.24)	65.29	<0.001	6.77 (4.26-10.8)
Location of metastases at diagnosis				
Only other sites	Reference			1.0
Only liver	0.11 (0.28)	0.16	0.69	1.12 (0.64-1.96)
Liver and other sites	0.05 (0.31)	0.03	0.88	1.05 (0.57-1.94)
Model 6: –2 log likelihood = 427.89				
WF stage				
IVa	Reference			1.0
IVb	0.87 (0.28)	9.55	0.002	2.39 (1.38-4.16)
IVc	1.63 (0.34)	22.37	<0.001	5.10 (2.60-10.0)
Lactate dehydrogenase				
<1.0 x UNL	Reference			1.0
1.0-2.0 x UNL	0.19 (0.21)	0.83	0.36	1.87 (0.80-1.83)
>2.0 x UNL	1.03 (0.33)	9.55	0.002	4.76 (1.46-5.37)

Abbreviations: CI, confidence interval; SE, standard error; UNL, upper normal limit; WF, Helsinki University Hospital Working Formulation stage.

References

[1] Moy CS, Albert DM, Diener-West M, McCaffrey LD, Scully RE, Willson JK. Cause-specific mortality coding: methods in the Collaborative Ocular Melanoma Study. COMS report no. 14. Control Clin Trials 2001; 22:248-62.
[2] Kujala E, Mäkitie T, Kivelä T. Very long-term prognosis of patients with malignant uveal melanoma. Invest Ophthalmol Vis Sci 2003; 44:4651-9.
[3] Eskelin S, Pyrhönen S, Hahka-Kemppinen M, Tuomaala S, Kivelä T. A prognostic model and staging for metastatic uveal melanoma. Cancer 2003; 97:465-75.
